# Myopathy associated BAG3 mutations lead to protein aggregation by stalling Hsp70 networks

**DOI:** 10.1038/s41467-018-07718-5

**Published:** 2018-12-17

**Authors:** Melanie Meister-Broekema, Rebecca Freilich, Chandhuru Jagadeesan, Jennifer N. Rauch, Rocio Bengoechea, William W. Motley, E. F. Elsiena Kuiper, Melania Minoia, Gabriel V. Furtado, Maria A. W. H. van Waarde, Shawn J. Bird, Adriana Rebelo, Stephan Zuchner, Peter Pytel, Steven S. Scherer, Federica F. Morelli, Serena Carra, Conrad C. Weihl, Steven Bergink, Jason E. Gestwicki, Harm H. Kampinga

**Affiliations:** 1 University Medical Center Groningen, University of Groningen, Department of Biomedical Sciences of Cell & Systems, Groningen, AV 9791 The Netherlands; 2 Institute for Neurodegenerative Disease, University of California at San Francisco, San Francisco, CA 94158 USA; 3 Department of Neurology, Washington University School of Medicine, St. Louis, MO 63110 USA; 4 Department of Neurology, Johns Hopkins School of Medicine, Baltimore, MD 21205 USA; 5 Programa de Pós-Graduação em Genética e Biologia Molecular, Department of Genetics, Universidade Federal do Rio Grande do SulPorto Alegre, Porto Alegre, 15053 Brazil; 6 Department of Neurology, Perelman School of Medicine, University of Pennsylvania, Philadelphia, PA 19104 USA; 7 Department of Human Genetics and Hussman Institute for Human Genomics, University of Miami, Miami, FL 33136 USA; 8 Department of Neuropathology, University of Chicago Pritzker School of Medicine, Chicago, IL 60637 USA; 9 Department of Biomedical, Metabolic and Neural Sciences, Center for Neuroscience and Nanotechnology, University of Modena and Reggio Emilia Modena, Modena, 41125 Italy; 10 Present Address: Max Planck Institute of Biochemistry, Martinsried/Munich, 82152 Germany

## Abstract

BAG3 is a multi-domain hub that connects two classes of chaperones, small heat shock proteins (sHSPs) via two isoleucine-proline-valine (IPV) motifs and Hsp70 via a BAG domain. Mutations in either the IPV or BAG domain of BAG3 cause a dominant form of myopathy, characterized by protein aggregation in both skeletal and cardiac muscle tissues. Surprisingly, for both disease mutants, impaired chaperone binding is not sufficient to explain disease phenotypes. Recombinant mutants are correctly folded, show unaffected Hsp70 binding but are impaired in stimulating Hsp70-dependent client processing. As a consequence, the mutant BAG3 proteins become the node for a dominant gain of function causing aggregation of itself, Hsp70, Hsp70 clients and tiered interactors within the BAG3 interactome. Importantly, genetic and pharmaceutical interference with Hsp70 binding completely reverses stress-induced protein aggregation for both BAG3 mutations. Thus, the gain of function effects of BAG3 mutants act as Achilles heel of the HSP70 machinery.

## Abstract (web summary)

BAG3 is a Hsp70 co-chaperone that is highly expressed in muscles. Here the authors show that several myofibrillar myopathy causing BAG3 mutations are not impaired in Hsp70 binding, but rather impair the ADP-ATP exchange step of the Hsp70 cycle, causing the aggregation of BAG3, Hsp70 and Hsp70 clients and leading to a collapse of protein homeostasis.

## Introduction

**Fig. 1:**
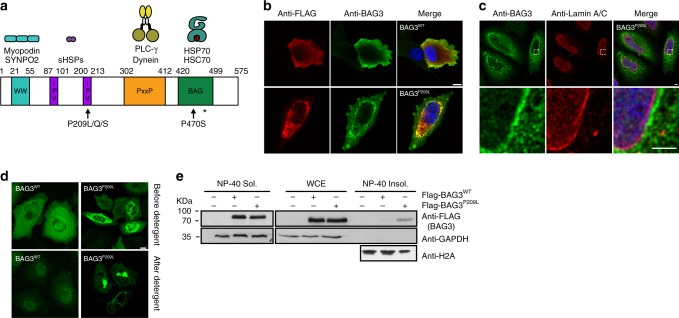
Cytoplasmic protein aggregation by BAG3P^209L^. **a** Schematic representation of BAG3 depicting the WW domain, the IPV motifs, the PxxP domain and the BAG domain. The disease-causing mutations P209L, P209Q, and P209S, and p470S are indicated with an arrow. The Hsp70-interaction disrupting mutation R480A is indicated with an *. **b** Immunofluorescence pictures of myoblast expressing FLAG-BAG3^WT^ or FLAG-BAG3^P209L^, using BAG3 (green) or FLAG (red) antibodies. DAPI staining is shown in blue. Scale bar = 5 μm. **c** Immunofluorescence pictures of HeLa cells expressing FLAG-BAG3^P209L^ using antibodies against BAG3 (green) and Lamin A/C (red). DAPI staining is shown in blue. Scale bar = 5 μm. **d** Immunofluorescence pictures of FLAG-BAG3^WT^ and FLAG-BAG3^P209L^ expressing HeLa cells using BAG3 antibody (green) before and after detergent treatment prior to fixation. Scale bar = 5 μm. **e** Whole cell extracts (WCE) and NP-40 soluble and insoluble fractions of HEK293 cells expressing indicated FLAG-BAG3 variants. Western blot against the indicated antibodies is shown. Source data are provided as a Source data file

BAG3 is a multi-domain scaffolding protein composed of a WW domain, two isoleucine–proline–valine (IPV) motifs, a PxxP domain, and a C-terminal BAG domain (Fig. 1a), enabling it to engage in multiple protein–protein interactions. It is the only stress-inducible BAG-family member and highly expressed in muscle^1^. Among the binding partners of BAG3 are different classes of molecular chaperones; it binds to 14-3-3 proteins^2,3^, to Hsp70 (also called HSPAs) via its C-terminal BAG domain, and to small heat shock proteins (also called HSPBs) via its IPV motifs (Fig. 1a)^1,4^. Together, these molecular chaperones play a crucial role in protein quality control (PQC)^5,6^. In this process, Hsp70s rely on co-chaperones of the DNAJ- and HSPB-families to deliver clients^7^, while nucleotide exchange factors (NEFs), including BAG3, promote client release^7^. BAG3 is thought to play a critical role because it can bind to Hsp70 and to HSPBs simultaneously^8,9^, thereby forming a ternary complex. This type of adapter activity is expected to be critical because HSPBs lack enzymatic function and are not able to refold clients; rather, they rely on collaboration with other ATP-driven chaperones, such as Hsp70s^10^. Thus, the proper timing and architecture of this multi-chaperone complex is likely extremely important to ensure a proper chaperone function. Interestingly, BAG3 is generally expressed at low levels in addition to being the only stress-inducible member of the BAG-family of NEFs. In fact, multiple types of stress can induce the expression of BAG3^11–13^, including mechanical stress as caused by muscle contraction^14–16^.

Several mutations in the individual components of the HSP70 machinery have been shown to cause disease^17^. Whereas no disease-associated mutations have been found in Hsp70 genes, suggesting that these may be incompatible with life, most so-called chaperonopathies, are caused by mutations in either *DnaJ* or *HspB* genes. The only two NEFs in which mutations are shown to cause disease are SIL1 and BAG3. Mutations in the *Sil1* gene, the ER-resident NEF, causes Marinesco-Sjögren syndrome, which is an autosomal recessive cerebellar ataxia associated with a myopathy characterized by vacuoles and protein inclusions^18^. Mutations in *Bag3* also cause a spectrum of disease phenotypes^1^. For example, a proline to leucine substitution at position 209 within the HSPB binding motif (referred to as BAG3^P209L^), causes a dominant, progressive myofibrillar myopathy (MFM) in which patients suffer from progressive muscle weakening, followed by sudden death due to heart failure in early adulthood^19^. A striking feature of this disease is the accumulation of protein aggregates in patient muscle, suggesting a collapse of protein homeostasis^19,20^. In addition, other missense mutations at the same P209 position (P209Q or P209S) within the IPV motif have been identified in multiple families with MFM and/or neuropathy^21,22^. Because these BAG3 mutations lie in the second IPV motif, loss of HSPB binding has been suggested as a molecular mechanism underlying disease^23,24^. However, in this study we report on a novel mutation in the BAG domain (BAG3^P470S^) as the cause of MFM, in two unrelated families. This observation calls into question whether the BAG3 mutants merely disrupt HSPB binding. Therefore, we set out to study the mechanism by which mutations in BAG3 protein drive such a devastating disease.

In contrast to the initial expectation, we show that a disturbed binding of BAG3 mutants to either HSPBs or Hsp70 does not underlie the dramatic collapse of PQC. In fact, for both the BAG3^P209^ and BAG3^P470^ mutants, binding to Hsp70 was found to be required for the phenotypic effects. These mutations caused aggregation of Hsp70 client protein and subsequent sequestration of PQC–proteins into insoluble aggregates. Importantly, the function of co-aggregating chaperones is severely hampered. Together, these events lead to a self-perpetuating progressive collapse of PQC. We found that the genetic or pharmacological disruption of the interaction between the mutant BAG3 proteins and Hsp70 is sufficient to revert this process. Thus, our data reveal how two different types of mutations in BAG3 lead to MFM in a similar manner that goes beyond a simple loss of function but is mediated by a dominant-gain of function on the Hsp70 chaperone machines. These findings suggest that BAG3 in particular, and maybe NEFs in general, are a potential Achilles heel of the Hsp70 machinery, where minor malfunctioning results in the entrapment of the whole chaperone complex with disastrous consequences for protein homeostasis. Inhibiting the BAG3–Hsp70 interaction using small molecules relieves this collapse and offers the first potential strategy to help these patients.

## Results

### HSPB binding is not linked to the BAG3^P209L^ phenotype

It is striking that several BAG3 mutations that lead to early onset MFM reside in the second IPV domain. To gain insight into what mechanism may underlay this, we initially focused on BAG3^P209L^ as a prime example of fulminant BAG3-related MFM. The BAG3^P209L^ mutation has been associated with the aggregation of sarcomeric proteins, including BAG3^P209L^ itself^19^. Whereas BAG3^WT^ was found to be distributed homogenously throughout the cell, ~50% of myoblast-cells expressing BAG3^P209L^ showed a punctae-like distribution pattern (Fig. 1b; Supplementary Figure 1a, b). Similar punctae were observed in HeLa and HEK293 cells upon ectopic expression of BAG3^P209L^ but not of BAG3^WT^ (Fig. 1c, d). Importantly, BAG3^P209L^ punctae appeared very similar in all cell types and were reminiscent of cellular aggregates observed in patients with BAG3^P209L^-associated MFM/DCM^19^. Punctae were strictly cytoplasmic (Fig. 1c and Supplementary Movie 1 for a 3D visualization) and resistant to pre-fixation extraction with detergent (Fig. 1d). Indeed, a significant portion of ectopically expressed BAG3^P209L^ is detergent insoluble in human cells, whereas BAG3^WT^ is primarily detergent soluble (Fig. 1e).

**Fig. 2:**
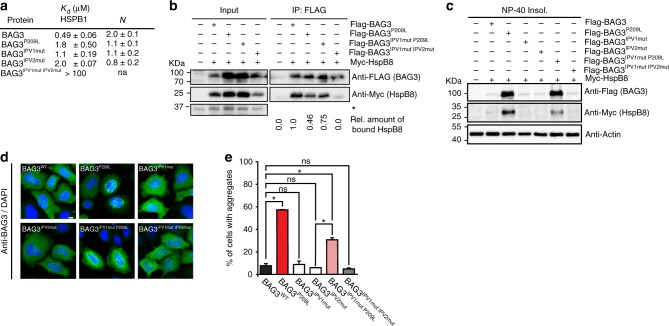
Protein aggregation by BAG3P209L is not caused by a loss of HSPB binding. **a** Binding of BAG3 to HSPB1c (Hsp27c) is partially disrupted by P209L. Binding affinity and estimated stoichiometry were measured by ITC. Results are the average of at least three independent experiments and error is standard deviation (SD). **b** Immunoprecipitation from HEK293 cells expressing FLAG-BAG3^WT^ or mutant variants using anti-FLAG beads. Western blots for FLAG (BAG3) and MYC (HSPB8) is shown. * indicates a cross-reactive band used as loading control. **c** NP-40 insoluble fraction of HEK293 cells expressing indicated FLAG-BAG3 variants. Western blot against the indicated antibodies is shown. The soluble fraction can be found in supplemental figure S2B. **d** Immunofluorescence pictures of HeLa cells expressing FLAG-BAG3^WT^ or indicated mutants, using a BAG3 antibody (green). Scale bar = 5 μm. **e** Quantification of the percentage of cells with BAG3 aggregates expressing the indicated variants of BAG3. Data represent the mean and standard deviation of two independent experiments (at least 100 cells were counted per experiment, Welch *t*-test was used to calculate the *P* values, * indicates *P* value < 0.05 and ns is not significant). Source data are provided as a Source data file

Our first hypothesis was that the P209L mutation might prevent binding of BAG3 to HSPBs because it is located in the second IPV motif^23–25^ (Fig. 1a). To test this idea, we measured the interaction of BAG3^WT^ and BAG3^P209L^ with a model, human HSPB fragment (HSPB1c/Hsp27c) using isothermal titration calorimetry (ITC). We found that Hsp27c binding to BAG3^P209L^ is indeed reduced 3.6-fold as compared to BAG3^WT^ (Fig. 2a). This loss of affinity was similar to the complete deletion of the IPV2 motif, but less pronounced than the deletion of both IPV motifs (Fig. 2a). Further, the estimated stoichiometry (N) of the complex was reduced from ~2:1 to ~1:1, which is consistent with the loss of one IPV interaction. To test if this loss of affinity was sufficient to affect BAG3-HSPB8 interactions in cells, we performed IPs and found that BAG3^P209L^ binding to HSPB8 was only mildly reduced (Fig. 2b). Rather, it took mutation of both IPV-motifs (BAG3^IPV1mut,IPV2mut^) to prevent the interaction. Mutating the first IPV motif in the BAG3^P209L^ background (BAG3^IPV1mut,P209L^) did not further affect HSPB8-binding, supporting the idea that the P209L substitution can still partially interact with HSPBs (Fig. 2b).

Intriguingly, a complete loss in HSPB binding (BAG3^IPV1mut, IPV2mut^) did not affect BAG3 solubility (Fig. 2c; Supplementary Figure 2a, b) and did not cause formation of BAG3 punctae (Fig. 2 d, e). Further going against expectations, we noticed that mutating IPV1 in the presence of the P209L mutation (BAG3^IPV1mut, P209L^) only modestly reduced punctae formation and improved solubility (Fig. 2 c–e; Supplementary Figure 2a, b). Together, these results surprisingly contradicted the initial hypothesis and, instead, support the idea that loss of HSPB binding is insufficient to cause the cellular BAG3^P209L^ phenotype.

### Recombinant BAG3^P209L^ forms non-native oligomers

**Fig. 3:**
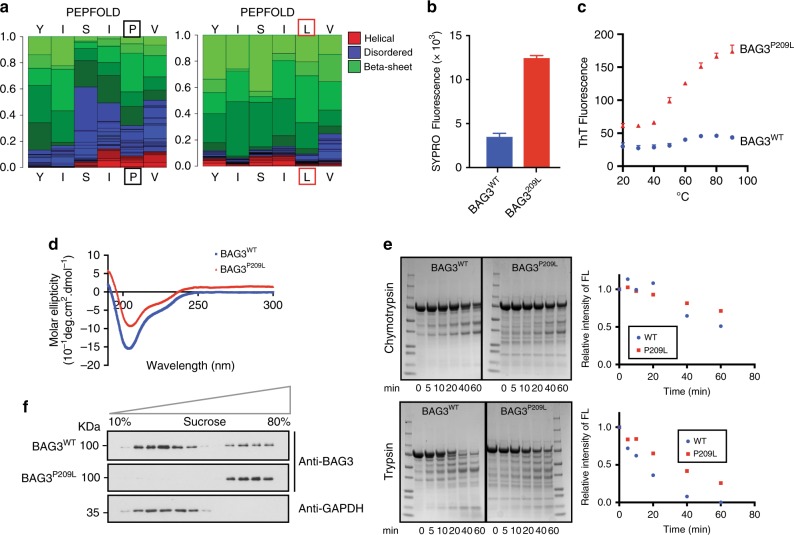
BAG3^P209L^ forms soluble oligomers. **a** Replacing proline for leucine at residue 209 is predicted to increase local beta-sheet content. PepFold was used to estimate propensity to form secondary structure. **b** Solutions containing recombinant BAG3^wt^ or BAG3^P209L^ were stained with SYPRO orange and fluorescence was measured. Results are the average of at least three independent experiments performed in triplicate each. Error bars represent SD. **c** Recombinant BAG3^wt^ or BAG3^P209L^ solutions were labeled by the beta-sheet dye, thioflavin T. Results are the average of at least three independent experiments performed in triplicate each. Error bars represent SD. **d** BAG3^P209L^ does not have gross changes in secondary structure, as estimated from CD measurements. Results are representative of measurements performed in triplicate. **e** BAG3^wt^ or BAG3^P209L^ solutions were subjected to partial proteolysis with either chymotrypsin or trypsin and samples were loaded on SDS–PAGE for separation. Coomassie stained gels are shown. **f** Fractionation of HEK293 cells expressing FLAG-BAG3^wt^ or FLAG-BAG3^P209L^ over a sucrose gradient. Western blot against BAG3 and GAPDH is shown. Source data are provided as a Source data file

Next, we considered a model in which the P209L mutation makes the BAG3 protein prone to aggregation. Using PepFold predictions, we had noticed that substituting leucine for proline at amino acid 209 was predicted to switch the local secondary structure from random coil to β-sheet (Fig. 3a). In line with this prediction, purified BAG3^P209L^ showed a marked increase in Sypro orange staining (Fig. 3b), indicative of exposed hydrophobic regions. Also, BAG3^P209L^—but not BAG3^WT^—showed a temperature-dependent increase in staining with the fluorescent β-sheet dye thioflavin-T (Fig. 3c). To further explore this relative instability, we measured the melting temperature of BAG3^WT^ and BAG3^P209L^ by differential scanning fluorimetry (DSF) and found that BAG3^P209L^ was nearly 4 °C less stable (BAG3^WT^ 49.6 °C ± 0.78 versus BAG3^P209L^ 46 °C ± 0.58). Strikingly, other hydrophobic mutations at position 209, such as Ile and Trp, were also predicted to increase β-sheet content (Supplementary Figure 3a), and the purified mutants (BAG3^P209I^ and BAG3^P209W^) were thioflavin-T positive (Supplementary Figure 3b). In contrast, mutations predicted in Pepfold to be random coil in this region such as BAG3^P209A^, BAG3^P209D^, BAG3^P209G^, and BAG3^P209K^ (Supplementary Figure 3a), were not thioflavin-T positive (Supplementary Figure 3b). Like BAG3^P209L^, the other mutants had reduced affinity for Hsp27c (Supplementary Figure 3c), consistent with loss of the second IPV motif. In summary, hydrophobic substitutions at the 209-position seemed to create a local, non-native structure.

Although these results initially suggested that BAG3^P209L^ might form insoluble aggregates, we found that recombinant BAG3^P209L^ is soluble under a wide range of conditions and that its overall fold is indistinguishable from BAG3^WT^ by circular dichroism (CD) (Fig. 3d). However, in SEC-MALS experiments, BAG3^WT^ and all control mutants (BAG3^P209A^, BAG3^P209D^, BAG3^P209G^, and BAG3^P209K^) were monomeric (~62 kDa), whereas BAG3^P209L^ and the hydrophobic mutants (BAG3^P209I^ and BAG3^P209W^) primarily formed soluble, higher order oligomers (Supplementary Figure 3d). Indeed, BAG3^P209L^ showed partial resistance to trypsin digestion and a modest resistance to chymotrypsin digestion (Fig. 3e), further suggesting a higher order structure. Consistent with these in vitro data, we separated detergent-soluble cell lysates on sucrose gradients and found that BAG3^P209L^ sediments at higher densities than BAG3^WT^ (Fig. 3f). Together, these results show that BAG3^P209L^ itself is not prone to forming insoluble aggregates, but that it does have a non-native quaternary structure.

### BAG3^P209L^ is impaired in stimulating HSP70-ATPase activity

**Fig. 4:**
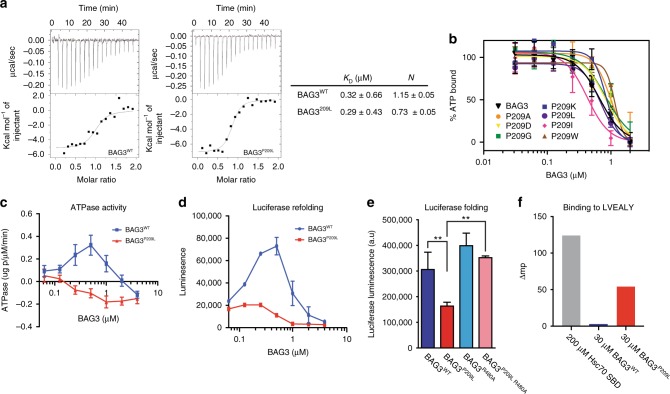
BAG3^P209L^ fails to collaborate with Hsp70 in client refolding. **a** Recombinant BAG3^P209L^ and BAG3^wt^ have normal affinity to HSPA8^NBD^. **b** All of the BAG3 variants are capable of releasing fluorescent nucleotide from Hsc70/HSPA9. Results are the average of at least three experiments performed in triplicate. Error bars represent SD. See the methods for details. **c**, **d** Recombinant BAG3^P209L^ is not functional in promoting HSPA8 steady state ATP hydrolysis (**c**) and Hsp70-mediated refolding assays (**d**). Measurement of ATPase activity and denatured luciferase refolding was carried out in the presence of Hsc70, DnaJA2, and various concentrations of BAG3^WT^ or BAG3^P209L^. Results are the average of at least three independent experiments performed in triplicate each. Error bars represent SD. **e** luciferase folding capacity of HEK293 cells expressing HSPB8 and BAG3^WT^ or indicated mutants of BAG3. Data represents the mean and standard deviation of two independent experiments (with three technical repeats for each experiment, Welch *t* test was used to calculate the *P* values, ** indicates *P* < 0.01). **f** Noncanonical interaction of BAG3^P209L^ with LVEAVY amyloid peptide. Source data are provided as a Source data file

We next wondered how the soluble BAG3^P209L^ oligomers might affect its interactions with Hsp70. In vitro and in cells, BAG3 and Hsp70 are known to coordinate cycles of client-binding and client-release, creating the delicate balance required for refolding^26,27^. To probe for a putative effect of the P209L mutation on its functional interaction with Hsp70, we initially measured the affinity of BAG3^WT^ and BAG3^P209L^ for the nucleotide-binding domain (NBD) of a representative Hsp70 family member, HSPA8, by ITC. Under these conditions, both BAG3^WT^ and BAG3^P209L^ bound normally to HSPA8^NBD^ (*K*_D_ ~ 0.3 µM) (Fig. 4a). Further, all of the point mutants had normal activity in releasing fluorescent nucleotide from HSPA8^NBD^ (Fig. 4b). Together, these data imply that, despite their nonnative quaternary structure, all of the mutants can bind to Hsp70.

Next, we wondered whether the BAG3^P209L^ oligomerization might have an impact on more complex, biochemical functions. For example, BAG3 transiently interacts with Hsp70s, such as HSPA8, during steady state ATP cycling. We found that BAG3^WT^ shows the expected concentration-dependent bell-shaped curve for stimulating the ATPase activity of HSPA8, characteristic for all BAG proteins (Fig. 4c)^28^. Despite the similar binding affinity, BAG3^P209L^ was inactive in these ATPase assays (Fig. 4c). This loss of activity of BAG3^P209L^ was further exacerbated at higher temperatures, consistent with the ThT and DSF assays. Thus, BAG3^P209L^ oligomers, despite being able to bind Hsp70, seemed to have a defect in coordinating its function over time. To investigate the consequence of this defect, we used denatured firefly luciferase as a model client protein. We first replicated the known behavior of BAG3^WT^, in which low levels (~0.2 µM) of the co-chaperone enhance the yield of folded luciferase by ~fourfold, while this stimulatory activity is lost at higher concentrations (Fig. 4d), mirroring the effect of BAG proteins in the Hsp70-ATPase assays (see Fig. 4c). Strikingly, BAG3^P209L^ was severely compromised in this assay, only inhibiting the reaction (Fig. 4d). To test whether the Hsp70–BAG3^P209L^ axis might be disrupted in living cells as well, we expressed BAG3^WT^ or BAG3^P209L^ and measured overall cellular folding capacity. It is known that over-expression of NEFs, such as BAG3, reduces the cellular luciferase folding capacity^29^, likely because NEFs are constitutively expressed at or near their optimal concentration (e.g., the equivalent of 0.2 µM in Fig. 4d). Despite this difficult backdrop, we found that elevated expression of BAG3^P209L^ reduced Hsp70-mediated folding capacity even further than high levels of BAG3^WT^ (Fig. 4e). In summary, hydrophobic mutations in BAG3 at position 209 induce a mild temperature dependent local unfolding that still allows proper Hsp70 binding but stalls the Hsp70 cycle at the client release stage.

### Disruption of Hsp70–BAG3^P209L^ binding rescues aggregation

**Fig. 5:**
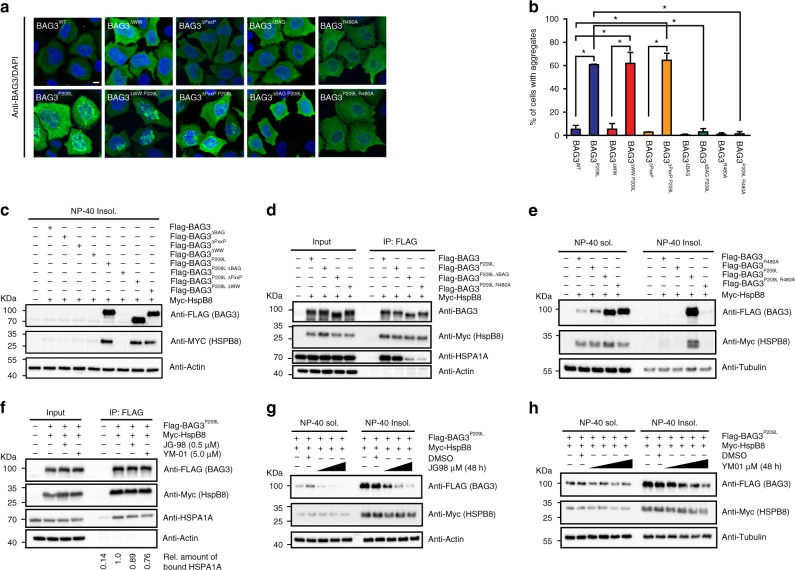
BAG3^P209L^ aggregation requires an interaction with Hsp70. **a** Immunofluorescence images of HeLa cells expressing FLAG-BAG3^wt^ and indicated mutants using an antibody recognizing BAG3 (green). Scale bar = 5 μm. **b** Quantification of the fraction of HeLa cells expressing the indicated BAG3 variants with immunofluorescence detectable punctae (2 experiments, at least 100 cells were counted per experiment, error bars represent SD, Welch *t* test was used to calculate the *P* values, * indicates *P* < 0.05). **c** Western blot of the NP-40 insoluble fraction of HEK293 cells expressing BAG3^wt^ and indicated variants of BAG3. **d** Immunoprecipitation of FLAG-BAG3 variants from HEK293 cells expressing both Myc-HSPB8 and indicated FLAG-BAG3 variants. Western blots using the indicated antibodies is shown. **e** Western blot of NP-40 soluble and insoluble fractions of HEK293 cells expressing indicated BAG3 variants; FLAG (BAG3), Myc (HSPB8), and tubulin antibodies were used. **f** Western blot of immunoprecipitates using anti FLAG beads from HEK293 cells expressing both FLAG-BAG3^P209L^ and Myc-HSPB8 in cells treated with either 0.5 μM JG-98, 5μM YM-01 or DMSO; FLAG (BAG3), Myc (HSPB8), HSPA1A, and actin antibodies were used. **g**, **h** Western blot of NP-40 soluble and insoluble fractions of HEK293 cells expressing both FLAG-BAG3^P209L^ and Myc-HSPB8, treated with DMSO or increasing concentration of the drug JG-98 (0.25, 0.5, or 1.0 µM) (**g**) or YM-01 (1.25, 2.5, 5.0, or 10 µM) (**h**). Source data are provided as a Source data file

Next, we wondered whether the failure to coordinate with Hsp70 might underlie the aggregation phenotype in cells. Therefore, we deleted the BAG domain, which is required for Hsp70 binding^9,28^, from BAG3^WT^ and BAG3^P209L^ and tested whether these proteins affect aggregation in cells. As controls, we also deleted the WW- and PxxP-domains, which are not involved in Hsp70 binding. When over-expressed in cells, none of the BAG3^WT^ deletions (BAG3^WT−∆WW^, BAG3^WT−∆PxxP^, or BAG3^WT−∆BAG^) resulted in punctae formation or insolubility (Fig. 5a–c). Also, expression of the BAG3^P209L−∆WW^ and BAG3^P209L−∆PxxP^ double mutants yielded punctae that were indistinguishable from those caused by BAG3^P209L^ single mutant alone (Fig. 5a–c). These results suggest that interactions with BAG3-binding partners, such as synaptopodins (WW domain)^1^ and SH3 domain-containing proteins, such as dynein motors (PxxP-motif)^1^, are not involved. Moreover, loss of Hsp70 interaction alone (BAG3^WT−∆BAG^) is not sufficient to cause an obvious aggregation phenotype. However, abrogating the ability of BAG3^P209L^ to interact with Hsp70 (BAG3^P209L−∆BAG^), fully prevented punctae formation and loss of solubility (Fig. 5a–c). Immunoprecipitation experiments confirmed that BAG3^P209L−∆BAG^ could no longer bind Hsp70s (Fig. 5d). These results confirm our in vitro data, showing that BAG3^P209L^ forms non-native oligomers, but that its interaction with Hsp70 is required for the formation of visible, BAG3^P209L^-related punctae.

The requirement of Hsp70 interaction for BAG3^P209L^-mediated aggregation was confirmed by experiments with a single point mutation, R480A, in the BAG domain (indicated with an * in Fig. 1a). This BAG3^R480A^ mutant is strongly impaired in Hsp70 binding (Fig. 5d) but is otherwise normal^28^. When expressed in cells, the R480A mutation could suppress the effects of P209L, as the double mutant (BAG3^P209L, R480A^) is neither insoluble (Fig. 5e), nor localized in punctae (Fig. 5a, b). Further, BAG3^P209L, R480A^, unlike BAG3^P209L^, did not have an adverse effect on the folding capacity in cells (Fig. 4e). To independently test this idea, we treated BAG3^P209L^ expressing cells with YM01 and JG98 molecules, which pharmacologically impair binding of Hsp70 to BAG3^30^. YM01 and JG98 reduce the affinity of Hsp70 to BAG proteins by trapping the ADP-bound state, resulting in a partial inhibition (Fig. 5f). Consistent with the double mutant data, both compounds reduced the BAG3^P209L^-associated insolubility in a dose dependent manner (Fig. 5g, h). Together, these results support the idea that protein aggregation caused by BAG3^P209L^ requires the recruitment of Hsp70. Thus, the slight decrease in BAG3^P209L^ stability (Supplementary Figure 3d and Fig. 3e) seems to be insufficient to induce aggregation by itself in vitro (Fig. 3e) or in cells (Fig. 5). Rather, upon binding to Hsp70, BAG3^P209L^ oligomers are recruited, along with Hsp70 clients. Then, because BAG3^P209L^ is unable to stimulate Hsp70-mediated function, we speculate that this complex stalls and eventually causes co-aggregation. It is difficult to monitor this type of higher order process in vitro, but we found that recombinant BAG3^P209L^ (but not BAG3^WT^) binds unfolded Hsp70 clients such as the LVEAVY peptide from IAPP (Fig. 4f), possibly via its non-canonical interaction surface^28^. Thus, we envision that multivalent interactions between BAG3^P209L^, Hsp70s, HSPBs and clients, driven by aberrant BAG3^P209L^-client contacts and avidity effects through multiple BAG domain-Hsp70^NBD^ contacts may disrupt dynamic protein-protein interactions and trigger aggregation.

### BAG3^P209L^ co-aggregates proteasomal substrates

**Fig. 6:**
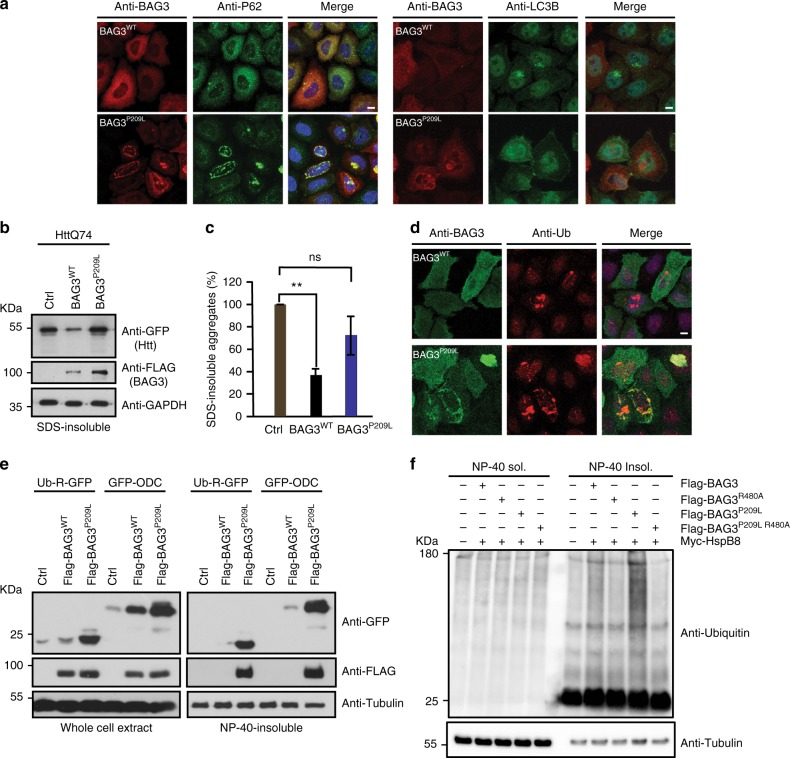
BAG3^P209L^ aggregation leads to co-aggregation of proteasomal substrates. **a** Immunofluorescence pictures of HeLa cells expressing FLAG-BAG3^WT^ or FLAG-BAG3^P209L^ using antibodies against BAG3 (green) or ubiquitin (red). Scale bar = 5 μm. **b** Suppression of GFP-HttQ74 aggregation of cells expressing a control, FLAG-BAG3^WT^ or FLAG-BAG3^P209L^. Western blot against indicated antibodies is shown. **c** Quantification of GFP-HttQ74 aggregation of experiments similar to **b**. Relative percentage of SDS-insoluble protein levels are shown. Data represents the mean and standard deviation of three independent experiments. **d** Immunofluorescence pictures of HeLa cells expressing FLAG-BAG3^WT^ or FLAG-BAG3^P209L^ using antibodies against BAG3 (green) or ubiquitin (red). Left column BAG3, middle column ubiquitin and right column is the merge of BAG3 (green), ubiquitin (red), and DAPI (blue). Scale bar = 5 μm. **e** Fractionation of HEK293 cells expressing HSPB8, a control or BAG3^WT^ or BAG3^P209L^, together with either Ub-R-GFP or GFP-ODC (ornithine decarboxylase). Western blot against GFP, FLAG (BAG3), Myc (HSPB8), and tubulin are shown. **f** Fractionation of HEK293 cells expressing HSPB8 and indicated BAG3 variants. Western blot using ubiquitin (FK2) and tubulin antibodies are shown. The same samples as in Fig. 5e have been used, loading control is therefore the same. Source data are provided as a Source data file

BAG3 has been found to be upregulated by various forms of stress that overload the proteasomal capacity^11,14^ and to redirect ubiquitinated Hsp70-clients from the proteasome to the autophagy-lysosome system^11,31^. During this process, termed BAG-induced proteasome to autophagy switch and sorting (BiPASS), BAG3 and Hsp70, along with ubiquitinated clients, temporarily localize to p62/LC3-positve punctae before being degraded by autophagy^11^. Intriguingly, the punctae formed by BAG3^P209L^ under nonstress conditions stain positive for p62, but not LC3B (Fig. 6a), suggesting that, although BAG3^P209L^ remains partially functional in initiating BIPASS, the process is somehow impaired or stalled. Indeed, compared to BAG3^WT^, the ability of BAG3^P209L^ to support the autophagic disposal of protein aggregates initiated by polyglutamine (polyQ) was much reduced (Fig. 6b, c; Supplementary Figure 4a)^4^. This result indicates that delivery of cargo to the autophagic system is impaired in BAG3^P209L^-expressing cells. In-line with this hypothesis, BAG3^P209L^ expression led to the accumulation of ubiquitin-positive punctae that co-localized with BAG3^P209L^ (Fig. 6d; Supplementary Figure 4b). Moreover, NP40-insoluble ubiquitinated proteins accumulated upon BAG3^P209L^-expression (Supplementary Figure 4c)^11^. To further investigate whether BAG3^P209L^ affects the disposal of proteasomal clients, we co-expressed the proteasomal reporters Ub-R-GFP (degraded following proteasome via the N-end-rule pathway^25^) and GFP-ODC (delivered to the proteasome via ubiquitin-independent pathways)^32^ with BAG3^WT^ or BAG3^P209L^. Expression of BAG3^WT^ led to small increases in both reporters (Fig. 6e), which reflects its role in rerouting proteasomal HSP70-clients to autophagosomes^11^. Mirroring its effects on endogenous ubiquitin, BAG3^P209L^ dramatically increased the levels of both reporters, revealing a dramatic loss in the cellular capacity to degrade proteasomal clients. Further, BAG3^P209L^, but not BAG3^WT^, led to the insolubilization of both proteasomal reporters (Fig. 6e). Experiments using a cell line stably expressing the proteasomal reporter Ub-G76V-YFP, which is degraded via the UFD-pathway^32^, showed qualitatively similar results (Supplementary Figure 4d). Expression of BAG3^IPV1mut, IPV2mut^ did not lead to the insolubilization of ubiquitinated proteins or their accumulation in BAG3^P209L^ punctae, while BAG3^IPV1mut, P209L^ led to less insolubilization and punctae formation of ubiquitinated proteins than BAG3^P209L^ (Supplementary Figure 4e, f), again showing that loss of HSPB binding is not responsible for the functional defect in BAG3^P209L^. Also, in line with our previous results (Fig. 5), Hsp70 binding is essential for the dominant defect evoked by expression of BAG3^P209L^: besides abrogating the BAG3^P209L^-insolubilization itself (Fig. 5), deletion of the BAG domain (but not the WW or PxxP domain) in the background of the P209L mutation also negated the BAG3^P209L^ effects on the insolubilization of ubiquitinated proteins (Supplementary Figure 4g) and their accumulation into the BAG3^P209L^ punctae (Supplementary Figure 4b). In addition, the BAG3^P209L,R480A^ double mutant neither led to the insolubilization of ubiquitinated proteins (Fig. 6f), nor to the accumulation and precipitation of the UFD reporter (Supplementary Figure 4d). Finally, JG98 or YM01 treatment counteracted the effects of BAG3^P209L^ on the insolubilization of ubiquitinated proteins (Supplementary Figure 4h, i). Together, these results indicate that BAG3^P209L^ leads to a general impairment of processing and aggregation of a wide range of ubiquitin-tagged client proteins of HSPB or/and HSP70.

### PQC proteins co-aggregate with BAG3^P209L^

**Fig. 7:**
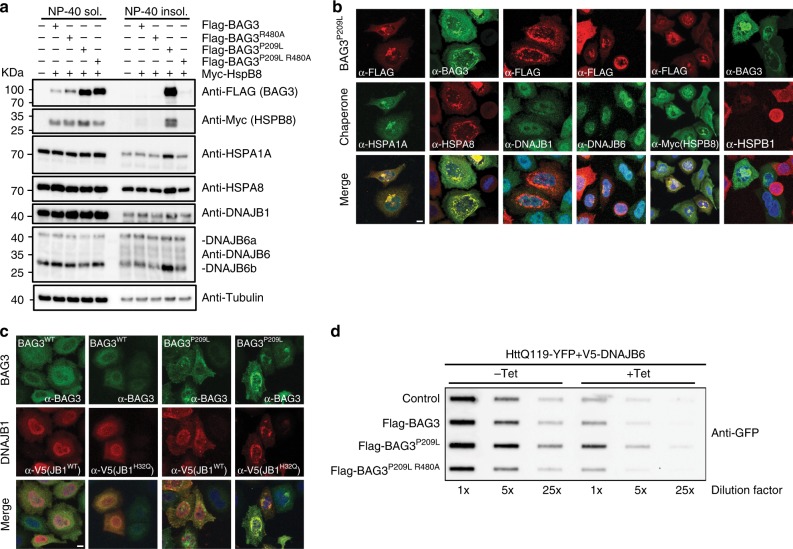
PQC proteins co-aggregate with BAG3^P209L^. **a** Fractionation of HEK293 cells expressing Myc-HSPB8 and BAG3 variants. Western blots using the indicated antibodies is shown. The same samples as in Fig. 5e have been used, loading control is therefore the same. **b** Immunofluorescence pictures of HeLa cells expressing FLAG-BAG3^P209L^. Cells were stained with the indicated antibodies for staining endogenous chaperones except HSPB8 (which was stained using antibodies against myc after expression of myc-HSPB8). Scale bar = 5 μm. **c** Immunofluorescence pictures of HeLa cells expressing BAG3^WT^ or BAG3^P209L^ (upper row in green) with V5-DNAJB1^WT^ or V5-DNAJB1^H32Q^ (middle row in red). Lower row depicts the merge with DAPI. Scale bar = 5 μm. **d** Filter trap assay of HEK293 cells expressing HttQ119-YFP together with tetracyclin-inducible DNAJB6b and the indicated FLAG-BAG3 variants. Immunoblot using a GFP antibody is shown. Source data are provided as a Source data file

How the accumulation of aggregates in cells can lead to toxic effects has been a matter of dispute for many years and may involve several, perhaps parallel acting, events^5^. One consequence of aggregation is the sequestration of components of the PQC systems, which leads to a vicious and progressive decline in protein homeostasis that subsequently impairs many cellular functions and eventually results in cell death^5^. Indeed, upon expression of BAG3^P209L^, we found that in addition to ubiquitinated proteins (Fig. 6) several chaperones (HSPA1A, HSPA8, HSPB8, DNAJB1, and DNAJB6), albeit not all (HSPB5 and HSPA6), to be enriched in the same detergent-insoluble fraction as BAG3^P209L^ (Fig. 7a; Supplementary Figure 5a). The same chaperones also co-localized with BAG3^P209L^-punctae (Fig. 7b; Supplementary Figure 5b, c; note that HSPB1 is also colocalizing with BAG3^P209L^-punctae). This co-aggregation was also highly dependent on the BAG3^P209L^–Hsp70 interaction as disruption of the BAG domain abrogated the co-aggregation (Fig. 7a; Supplementary Figure 5a–c). Many DNAJs are known to have their own client-binding ability that is not dependent on Hsp70-interactions. We found that the recruitment of DNAJB1 and DNAJB6 into aggregates is independent of their interaction with Hsp70 as the DNAJB1^H32Q^ and DNAJB6^H31Q^ mutants still co-localized with BAG3^P209L^ punctae (Fig. 7c), suggesting that their co-localization with punctae is Hsp70 independent. One possibility is that co-chaperones are recruited by clients and trapped when they become insoluble. In line with such a trapping model, only the mainly cytosolic DNAJB6 isoform (DNAJB6b, 26 kDa) became insoluble in BAG3^P209L^-expressing cells, while the exclusively nuclear isoform (DNAJB6a, 40 kDa) remained soluble (Fig. 7a).

### Sequestration of chaperones suppresses their function

Trapping of (co-)chaperones could contribute to disease by reducing their effective pool in the cell. To test this idea, we measured the ability of DNAJB6b to suppress polyQ aggregation in the background of BAG3^WT^ or BAG3 mutants. BAG3^WT^ can reduce aggregation initiated by poly_Q74_ expansions (Fig. 6)^4^ but not aggregation initiated by extremely long poly_Q119_ fragments, while DNAJB6 co-expression alone almost completely suppresses poly_Q119_ aggregation (Fig. 7d)^33,34^. In line with the hypothesis of a dominant negative effect via sequestration of other chaperones into BAG3^P209L^ aggregates, co-expression of BAG3^P209L^—but not BAG3^WT^ or BAG3^P209L, R480A^—inhibited DNAJB6-mediated anti-aggregation (Fig. 7d). Together, these data reveal that BAG3^P209L^ directly stalls the Hsp70 function, which—as a result—perpetuates into a dominant effect on the function of other chaperones.

### Stalling of HSP70 in other BAG3-related MFM mutations

**Fig. 8:**
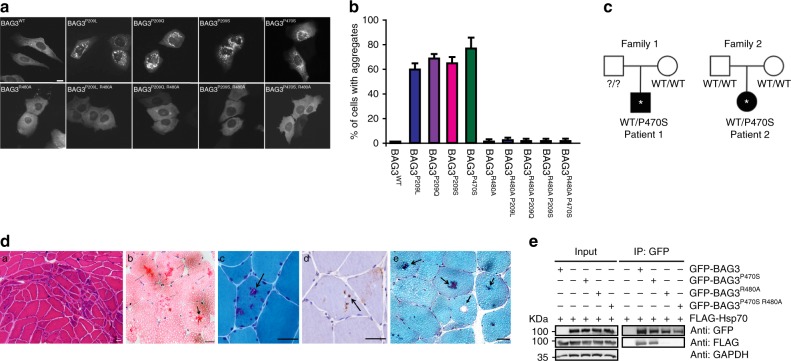
MFM-causing BAG-domain mutations also cause HSP70-dependent aggregation. **a** Immunofluorescence pictures of HeLa cells expressing the indicated BAG3-disease causing mutants, with or without the additional R480A mutation that abrogates the ability of the (mutant) BAG3s to interact with Hsp70. Scale bar = 5 μm. **b** Analyses of the number of cells expressing the indicated BAG3-disease causing mutants (with or without the additional R480A mutation that abrogates their ability BAG3s to interact with Hsp70) that contain over 20 punctae. The increase in cells with punctae in the single mutant backgrounds and their abrogation in the double mutant backgrounds are significant with a *P* value of <0.00005 (*t* test). **c** Pedigree structures and known genotype of the proband (*) and parents in Family 1 and Family 2 with a P470S mutation in the BAG-domain. **d** Histochemical and immunohistochemical analysis of muscle biopsies from two novel MFM patients carrying a P470S mutation in the BAG domain of BAG3: Patient 1 (**a**–**c**) and Patient 2 (**d**, **e**) (see Fig. S6B, C for further patient info); **a** hematoxylin and eosin (H&E) staining demonstrates region of grouped atrophy with small fibers harboring vacuoles; **b** modified gomori trichrome (GT) staining demonstrating both cytoplasmic inclusion and rimmed vacuoles; **c** immunohistochemistry with an antibody to TDP-43 demonstrates protein accumulation; **d** H&E staining shows large centrally located eosinophilic inclusion; **e** GT staining shows dark centrally located inclusions and a fiber with a centrally located vacuole. Arrows denote protein inclusions or vacuoles. Scale bar = 50 μm. **e** Immunoprecipitation of GFP-tagged BAG3 variants from cells expressing both GFP-BAG3 variants and FLAG-tagged HSP70. Western blots using the indicated antibodies is shown. Source data are provided as a Source data file

To test if impediment of Hsp70 functioning by single amino acid substitutions in BAG3 occurs more frequently, we turned to two other mutations at the P209 position, BAG3^P209Q^, and BAG3^P209S^ that cause MFM or neuropathy, respectively^21,22^. Like BAG3^P209L^, both BAG3^P209Q^ and BAG3^P209QS^ form multiple punctae when expressed in cells (Fig. 8a). Likewise, genetic abrogation of the Hsp70 interaction (BAG^R480A,P209Q^ BAG3^P209S,R480A^) reverts this phenotype as well (Fig. 8a, b), implying that all P209 disease-causing mutants operate via a similar dominant Hsp70-stalling mechanism.

In order to further understand BAG3 associated MFM pathogenesis and identify potentially novel BAG3 genetic variants that are associated with MFM, we performed exome sequencing on patients with muscle weakness and features of MFM on muscle biopsy. This approach identified two unrelated MFM patients carrying an unreported BAG3 c.1408C>T; p.P470S missense mutation (Supplementary Figure 6a). Neither patients had a family history of muscle weakness or other neuromuscular disorder. Whole exome sequencing of patient 2’s living parents failed to identify the BAG3 c.1408C>T; p.P470S variant, suggesting that it was a de novo mutation in this patient. It was only possible to obtain DNA samples from Patient 1’s mother, confirming that she was a nonvariant carrier (Fig. 8c). Muscle tissue from both patients demonstrated features characteristic for MFM patient tissue with sarcoplasmic inclusions and rimmed vacuoles as visualized on hematoxylin and eosin staining and gomori trichrome (Fig. 8d).

Amino acid P470 is conserved in all human BAG proteins, except BAG2, and amongst various BAG3 from other vertebrate species (Supplementary Figure 6b). The P470S variant is predicted to be damaging by Polyphen2 and Mutation Taster. Moreover, it is not found within public databases, including the EXAC browser and GNOMAD, further supporting its pathogenicity. Unlike the previously reported BAG3 mutations, which reside within the second IPV motif, the P470 residue lies within the HSP70 binding region or BAG domain. Since our proposed model of BAG3 mutant dysfunction suggested that mutations in the IPV motif destabilize the protein and alter HSP70 kinetics that require HSP70 binding, we tested whether the P470S missense mutation within the BAG domain has affected HSP70 binding as well. Surprisingly, the BAG3^P470S^ mutant maintained HSP70 association, albeit with a slight loss in binding, unlike the BAG3^R480A^ mutant that abolishes HSP70 binding (Fig. 8e). In addition, similar to BAG3^P209L^, BAG3^P209Q^, and BAG3^P209S^, BAG3^P470S^ aggregated in cell culture, which was corrected by a secondary R480A mutation (Fig. 8a, b). Together these results suggest that all MFM related BAG3-mutants tested require interaction with Hsp70 to become pathological and result in a dominant gain of impairment of the functions of Hsp70-related networks.

### Abrogation of BAG3–Hsp70 by pharmaceutical intervention

**Fig. 9:**
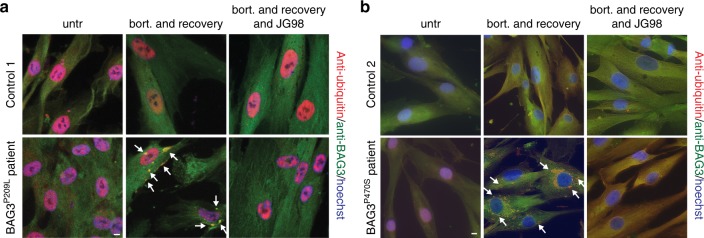
Abrogation of BAG3–Hsp70 by pharmaceutical intervention. **a**, **b** Immunofluorescence pictures using BAG3 (green) and ubiquitin (FK2; red) antibodies of primary control versus BAG3^P209L^ patient fibroblasts (**a**) or primary control versus BAG3^P470S^ patient fibroblasts (**b**). Cells were either untreated, treated for 6 h with bortezomib followed by a 20 h recovery or treated for 6 h with bortezomib followed by a 20 h recovery in the presence of 0.05 µM JG98. Arrows indicate BAG3-punctae. Scale bar = 5 μm. Source data are provided as a Source data file

To address whether endogenous, rather than over-expressed, BAG3 mutations results in similar phenotypes, we performed experiments with fibroblasts derived from control patients or patients carrying the BAG3^P209L^ and BAG3^P470S^ mutations. Endogenous expression of BAG3 is low in most cell types, including fibroblasts, which may be the reason why no spontaneous mutant BAG3-related punctae were observed in the patient fibroblasts (Fig. 9a, b). However, as stated above, BAG3 is the only stress-inducible BAG–protein^35^ and is upregulated by a variety of stress signaling cascades, including those initiated by treatment with proteasome inhibitors^11^. Indeed, a short treatment with bortezomib produced persistent ubiquitin-positive BAG3 punctae in fibroblasts from both BAG3^P209L^ and BAG^P470S^ carriers but not in fibroblasts from patients without the mutation (Fig. 9a, b). To test whether this punctae formation can be reversed by disrupting BAG3–Hsp70 interactions, we co-treated the BAG3 mutated, MFM patient-derived fibroblasts with bortezomib and the drug JG98 that disturbs Hsp70–BAG3 interactions (Fig. 5f) and reverts aggregates formation when the mutants are overexpressed (Fig. 5, Supplementary Figure 4). As predicted, this treatment indeed reduced the frequency of persistent BAG3 punctae in the lines from both BAG3^P209L^ and BAG^P470S^ carriers (Fig. 9a, b). This confirms that they cause aggregation via similar mechanisms and imply that pharmaceutically targeting the Hsp70–Bag3 interaction could be a first therapeutic strategy for patients carrying P209L/Q/S or P470S substitutions.

## Discussion

Mutations in BAG3 are associated with a devastating syndrome that includes MFM, dilated cardiomyopathy, and neuropathy. Why certain mutations result in a more severe clinical outcome is unclear. Thus far MFM was associated with mutations at P209 position that lies in the second IPV motif^19,36–38^. Here, we describe a second mutation site (P470S) that lies in the BAG domain. The notion that both are localized in domains that connect this central co-chaperone to other chaperone systems initially led us to hypothesize that the mutation might block binding to HSPBs or Hsp70, leading to loss-of-function as has been suggested. However, our results suggest a more nuanced mechanism characterized by only mildly impaired chaperone binding but a more intense outcome, i.e., a dramatic stalling of Hsp70 client processing via a disruption in their ability to functionally stimulates the ATP activity of HSP70s. In cells, these molecular defects subsequently result in the formation of protein aggregates via interactions with Hsp70. These aggregates next trap chaperones that become partly nonfunctional, which is exemplified by the BAG3^P209L^-effects on DNAJB6 function. Intriguingly, just like BAG3 mutations, mutations in DNAJB6 have been linked to myopathies as well^39,40^.

Interestingly, whereas absence or loss of function of BAG3 result in an increase in insoluble proteins^41^, this is not sufficient to cause the formation of large aggregates triggered by BAG3^P209L^, BAG3^P209Q^, BAG3^P209S^ or BAG3^P470S^ mutations. Apparently, loss (or partial loss) of BAG3 function does affect PQC, but dominant gain of function mutations, including the MFM-related BAG3 mutations, are more deleterious by triggering the negative spiral of trapping and co-aggregating of Hsp70-clients, Hsp70 itself, and other PQC components. The latter results in loss of function of other chaperones. Our data reveal a novel molecular mechanism that would explain the far more progressive and early onset aspects related to MFM pathology. This concept has potentially broad implications because multiple myopathies and neuropathies are caused by dominant mutations in PQC components as well.

It is also interesting to compare the BAG3-related diseases to other chaperonopathies, like those caused by recessive or dominant mutations in other Hsp70 co-chaperones such as DNAJ or HSPB proteins (Supplementary Figure 7a)^17^. Mutations in DNAJs or HSPBs are typically associated with late onset cardiomyopathies or neuropathies. In contrast, mutations in the NEFs (Sil-1 and BAG3) often cause early onset or congenital disease^18,19,22,36–38^. The reason for this is not known but could reside in the fact that DNAJs and HSPBs primarily select the input of clients into Hsp70 chaperones machines (Supplementary Figure 7b). This implies that only a certain number of substrates are affected. Moreover, the handling of these substrates can, at least for some time, be taken care of by alternative PQC mechanisms as has been suggested for DNAJB6 mutations^42^. In contrast, mutations in BAG proteins, as being NEFs, are responsible for the output of a larger range and number of substrates (Supplementary Figure 7b). Therefore, impairment of NEF functions like that of Sil1 and BAG3 may impede on more clients and thus have a more drastic impact. For, BAG3 loss of heterozygosity already leads to widespread and early onset diseases^19,22,36–38^. Even more so, BAG3 is involved in what already seems to be a back-up PQC mechanism that compensates for an overload of the proteasome (BIPASS)^11^.

The high vulnerability of skeletal and cardiac muscle cells to BAG3 mutations may be linked to the fact that BAG3 is constitutively expressed at relatively high levels in these tissues. Stress situations that overload the proteasome increase BAG3 levels without affecting expression of other members of the BAG family^35^, probably helping cells to reroute proteasomal clients to autophagosomes^11^. Muscle cells are repeatedly exposed to cellular stress (e.g., mechanic stress from exercise) that may elevate BAG3 expression to levels sufficient for the primary defects of mutants, like BAG3^P209L^, to start take effect. Among the BAG family members, BAG3 has the highest affinity for Hsp70s^27^, meaning that a stress-related imbalance in relative BAG levels may suffice to initiate the negative cascade described here. Indeed, the data on patient-derived fibroblasts provides a proof-of-concept for this idea.

These results suggest that either genetic or small molecule perturbation of the Hsp70–BAG3 interaction might not only attenuate the aggregation cascade but also minimize its toxic, downstream effects. This result suggests that the Hsp70–BAG3 contact could be a drug target for the treatment of BAG3-related myopathies. Indeed inhibitors of this interaction, have been advanced to preclinical safety studies in mice^27^. However, any such treatment for human myopathy would require long-term administration and the safety of disrupting a central chaperone activity is not yet clear. Nevertheless, our results suggest that further development of more specific inhibitors of the BAG3–Hsp70 interaction, for example ones that selectively target the mutant BAG3–Hsp70 interaction, may be promising.

## Methods

### Plasmid construction

Plasmids encoding myc-tagged human HSPB8^43^ and peGFP-HDQ74, were kindly provided by Dr. D.C. Rubinsztein^44^. FLAG-BAG3 WT and P209L constructs were kindly provided by Dr. S. Takayama. The primers and plasmids used in this study are listed in Supplementary Table 1 and Supplementary Table 2. To generate Flag tagged human BAG3 (pcDNA3 FLAG-BAG3), BAG3 delta BAG (pcDNA3 FLAG-BAG3^ΔBAG^), and BAG3 delta PxxP (pcDNA3 FLAG-BAG3^ΔPxxP^), the BAG3 encoding sequences of the following constructs were amplified by polymerase chain reaction (PCR) with Phusion polymerase, purified by agarose gel electrophoresis and ligated to EcoRI and XhoI digested pcDNA FLAG-Parkin: BAG3 (pCN His-BAG3), BAG3 ΔBAG (pCN His-BAG3^ΔBAG^) and BAG3 ΔPxxP (pCN His-BAG3^ΔPxxP^). Using specific primers, the P209L mutation was introduced into Flag-tagged BAG3 constructs with the *Pfu* turbo DNA polymerase site directed mutagenesis kit to generate the following constructs: BAG3P209L (pcDNA3 FLAG-BAG3^P209L^), BAG3 delta BAG P209L (pcDNA3 FLAG-BAG3^ΔBAG, P209L^), BAG3 delta PxxP P209L (pcDNA3 FLAG-BAG3^ΔPxxP, P209L^), BAG3 delta WW (pcDNA3 FLAG-BAG3^ΔWW^), and BAG3 delta WW P209L (pcDNA3 FLAG-BAG3^ΔWW, P209L^). BAG3 IPV1 (pcDNA3 FLAG-BAG3^IPV1>AAA^), BAG3 IPV2 (pcDNA3 FLAG-BAG3^IPV2>AAA^), BAG3 IPV1 P209L (pcDNA3 FLAG-BAG3^IPV1>AAA, P209L^), and BAG3 IPV1 IPV2 (pcDNA3 FLAG-BAG3^IPV1>AAA, IPV2>AAA^) were constructed in a similar fashion; all constructs were sequence verified. All domain deletion constructs used for in vitro studies were subcloned from the BAG3 pMCSG7 parent vector and confirmed with DNA sequencing. Mutations were constructed using standard mutagenesis protocols.

### Recombinant protein production

All constructs were transformed into BL21(DE3) cells and single colonies were used to inoculate TB medium containing ampicillin (50 μg/mL). Cultures were grown at 37 °C to an OD_600_ 0.6, when NaCl and betain were added to a final concentration of 500 and 10 mM, respectively. The cultures were cooled to 18 °C and induced overnight with 500 μM IPTG. BAG3-expressing cells were pelleted, resuspended in BAG3 lysis buffer (50 mM Tris, 100 mM NaCl, 1 mM EDTA, and 15 mM β-mercaptoethanol (pH 8.0)), microfluidized, and applied to Ni-NTA resin. After Ni-NTA columns, all proteins were subjected to TEV protease cleavage overnight and dialyzed into MonoQ buffer A (20 mM HEPES, 10 mM NaCl, and 15 mM β-Mercapthoethanol (pH 7.6)). Proteins were applied to a MonoQ column (GE Healthcare) and eluted by a linear gradient of MonoQ buffer B (buffer A + 1 M NaCl). Fractions were dialyzed into BAG buffer (25 mM HEPES, 5 mM MgCl_2_, and 150 mM KCl (pH 7.5)) and concentrated. Hsc70, Hsc70_NBD_, Hsc70_SBD_, J proteins, and Hsp27c were purified using a His column and subsequent SEC on a Superdex S200^9^. Further details are provided in the Supplementary Methods.

### DSF and ThT melt curve

Samples of BAG3^WT^ or BAG3^P209L^ in BAG3 buffer (20 μL samples of 0.3 mg/mL) with a 5× final concentration of SYPRO Orange (Sigma) were placed into a white 96-well plate with optically clear caps. DSF melt curves were acquired on a Stratagene Mx300P RT-PCR using the SYPRO filter set. Samples were heated from 25 to 95 °C in 1 °C increments, and the melting temperature was determined using a Boltzmann fit. For ThT melt curves, 100 μL samples of 10 μM BAG3^WT^ or BAG3^P209L^ with 10 µM ThT were heated in a thermocycler from 20 to 90 °C in 10 °C increments and removed from heating at each temperature. Samples were cooled to 20 °C and plated in a low volume, black 384-well plate (Corning) and ThT fluorescence was read with 444 nm excitation, 485 nm emission, and a 475 nm cutoff using a SpectraMax5 M5 multi-mode plate reader. All experimental data were analyzed using Prism 7.0 software (Graphpad Software).

### CD

BAG3^WT^ and BAG3^P209L^ were dialyzed overnight against 20 mM NaP_i_ with 100 mM NaF (pH 7.5). 0.1 mg/mL samples were placed in a low-volume (250 μL) cuvette. Measurements were taken at 25 °C using a Jasco710 instrument.

### Partial proteolysis

BAG3^WT^ or BAG3^P209L^ (0.8 mg/mL or ~13 μM) in BAG buffer was subjected to partial proteolysis with either chymotrypsin (1/5000 ratio BAG3/enzyme) or trypsin (1/10,000). The samples were incubated at 37 °C for specified time and reactions were quenched by adding sodium dodecyl sulfate (SDS) sample loading dye and boiling at 98 °C for 5 min. Protein fragments were separated by SDS–polyacrylamide gel electrophoresis (PAGE) and stained with Coomassie.

### ITC

BAG3 constructs, Hsp27c and Hsp72^NBD^ were dialyzed overnight against ITC buffer (25 mM HEPES, 5 mM MgCl_2_, 100 mM KCl [pH 7.5]). Concentrations were determined using a BCA Assay (Thermo Scientific), and the experiment was performed with a MicroCal microITC (GE Healthcare) at 25 °C. Hsp72^NBD^ (100 μM) or Hsp27c (200 μM) in the syringe was titrated into a 10 μM cell solution of BAG3 protein. Calorimetric parameters were calculated using Origin^®^ 7.0 software and fit with a one-site binding model.

### Antibodies and reagents

Antibodies (dilutions are indicated in brackets for western blot (WB), immunofluorescence (IF), or immunoprecipitation (IP)) against FLAG (Sigma, clone M2; Sigma, produced in Rabbit, IP 3 μl/sample, IF 1:100, WB 1:1000), FLAG (Sigma, clone M2; Sigma, M, Wb 1:1000, IF 1:200), ubiquityl-histone H2A (Millipore, clone E6C5), ubiquitin (Norvus Biologicals, FK2, M, WB 1:1000, IF 1:1000; Dako WB), K48-linkage specific polyubiquitin (Enzo lifesciences, WB 1:1000), K63-linkage specific polyubiquitin (Cell Signaling, clone D7A11, 1:1000), myc (MBL, clone PL14, WB 1:3000, IF 1:100), HSC70 (Stressgen, WB 1:5000, IF 1:100), LC3B (Novus Biologicals, NB100-220), GFP (clonetech, 632381), p62 (Enzo Life Sciences, BML-PW9860), Lamin A/C (Santa Cruz, 4A58), HSC70 (Stressmarq biosciences), HSP70 (Stressgen, clone SPA-810, WB 1:1000, IF 1:50), HSPA1A (Enzo life sciences, Rb, WB 1:1000), HSPB1 (Stressmarq biosciences), GAPDH (Fitzgerald, clone 6C5, WB 1:50,000), histone H2A (Abcam, WB 1:5000), MYC (Clonetech, Mountain View, CA, USA), and DNAJB1/Hsp40 (Stressgen, San Diego, CA, USA, Rb, 1:1000) were used.

MG132 (20 µM for 3–6 h), rapamycin, Pepstatin A (10 μg/ml), E64d (10 μg/ml), 3-methyladenine (3-MA, 10 mM) ammonium chloride (NH_4_Cl, 20 mM) were from sigma.

### Cell culture and transfection

Standard cell culture techniques were used. HeLa (human cervical cancer), HEK293, and HEK293T (human embryonal kidney) cells were grown at 37 °C and 5% CO_2_ in Dulbecco’s modified Eagle’s medium with high glucose supplemented with 10% fetal calf serum and 1% penicillin/streptomycin (Gibco). LHCNM2 human myoblasts were cultured in HAM’s F12, supplemented with 20% FBS, 2 mM l-glutamine, 100 U/mL penicillin/streptomycin and 25 ng/mL of rh FGF-b/FGF-2^45^. Cell lines stably expressing GFP-ubiquitin (kind gift of Dr. Dantuma) were generated by transfecting GFP-ubiquitin into HEK293-cells with lipofectamine using standard procedures. HEK293T and HeLa-cells were transfected with calcium phosphate precipitation, or with lipofectamine (Invitrogen) or polyethylenimine (PEI) (Sigma-Aldrich, St. Louis, MO, USA) according to manufacturer’s protocols.

### Chaperone assays

The steady-state ATPase activity of Hsc70 was measured by malachite green as previously reported^46^. For ATPase activity, Hsc70 (final concentration 1 µM), DnaJA2 (final concentration 0.5 µM) and various concentrations of BAG3 WT or P209L were added to clear 96-well plates to give a volume of 15 µL. The reaction was initiated by adding 10 μL of 2.5 mM ATP to give a final concentration of 1 mM. Plates were covered and incubated at 37 °C for 1 h. The reactions were developed with malachite green, quenched with sodium citrate, and plate absorbance was measured at 620 nm.

### Luciferase refolding assay

The refolding of chemically denatured firefly luciferase was measured by recovered luminescence^27^. Briefly, working stocks of denatured luciferase were prepared by mixing 10 μL of 200 μM native firefly luciferase (Promega) with 30 μL of 8 M GnHCl for 1 h at room temperature. Denatured luciferase stocks were stored at −80 °C until use. To white 96-well plates, was added denatured luciferase (final concentration of 100 nM), Hsc70 (final concentration of 1 µM), DnaJB4 (final concentration of 0.5 µM), and various concentrations of BAG3^WT^ or BAG3^P209L^ to give a final volume of 25 μL in refolding buffer (20 mM HEPES, 120 mM KAc, 1.2 mM MgAc, 15 mM DTT, 60 mM creatine phosphate, 35 U/mL creatine kinase, 5 ng/μL BSA, pH 7.4). The reaction was initiated by adding 10 μL of 2.5 mM ATP to give a final concentration of 1 mM. Plates were covered and incubated at 37 °C for 1 h. Finally, 25 μL of Steady-Glo reagent (Promega) was added to each well and luminescence values were measured immediately.

### SEC-MALS

Solutions of BAG3^WT^ or BAG3^P209L^ (30 µM) were separated by size exclusion chromatography (Shodex 804), as previously described^9^. Molecular weights were determined by multi-angle laser light scattering using an in-line DAWN HELEOS detector and an Optilab rEX differential refractive index detector (Wyatt Technology Corporation). Calculation of molecular weights was performed using the ASTRA software package (Wyatt Technology Corporation).

### Fluorescent peptide assay

Experiments were performed in 384-well, black, low volume, round-bottom plates (Corning) using a SpectraMax5 M5 multi-mode plate reader^47^. To each well, was added specified amount of protein and the 5-carboxyfluorescein (5-FAM) labeled LVEALY tracer (20 nM) to a final volume of 20 μL in the assay buffer (50 mM HEPES, 75 mM NaCl, 0.01% Triton X-100, pH 7.4). The plate was incubated at room temperature for 30 min to reach equilibrium. The polarization values in millipolarization units (mP) were measured at an excitation wavelength at 485 nm and an emission wavelength at 535 nm.

### Protein-pull down

For IP, cells were lysed on ice in IP lysis buffer 24 h posttransfection (buffer: 60/80 mM KCl, 50 mM HEPES pH 7.5, 1.5 mM MgCl_2_, 0.4% Nonidet P-40, 10/3% glycerol, 0.5 mM DTT, complete EDTA-free (Roche Applied Sciences) and 10 mM NEM). Cell lysates were homogenized by passing lysates 5/6 times through a 26 G needle and the total or “whole cell lysate” was collected. Lysates were centrifuged at maximum speed for 15 min at 4 °C in order to separate the supernatant (input or soluble fraction) from the pellet fraction (insoluble fraction). The protein concentration was determined (using DC^™^ protein assay kit from Bio-Rad) and input fractions were equalized. In the meantime, magnetic beads complexed with FLAG-antibodies (14 μl beads/sample; Sigma, FLAG M2, clone) were washed with IP lysis buffer. Pellet fractions were resolubilized with 2% SDS buffer containing beta-mercapthoethanol, boiled, and stored for future use. Co-IP was performed by adding equal amounts of input fraction onto the washed magnetic beads and incubating them at slow rotation (5 RPM) for at least 2 h at 4 °C. The first wash was performed using the IP-volume in lysis buffer and a magnetic stand. In between washes, beads were incubated on the rotator for 5 min at slow rotation at 4 °C. Further three washes were performed in a similar way using 1 mL of IP lysis buffer. During the last wash, beads were transferred into new cups and washed with IP lysis buffer without detergent. Proteins were eluted using ½ volume 2% SDS buffer and ½ volume 4 × 2% SDS buffer and 10% beta-mercapthoethanol. Samples were kept on ice until they were boiled for 5 min co-immunoprecipitated proteins and input fractions were resolved on SDS-PAGE the same day as the IP was performed.

### Fractionations

Protein measurement was performed using the nanophotometer (Implen), protein content was equalized and equal amounts of volume and protein were used for subsequent fractionation. SDS fractionation was performed as previously described^34,48^. For NP40 fractionations, cell-pellets were resuspended in NP40 fractionation buffer (fractionation buffer: 50mM HEPES, pH 7.4, 2mM MgCl_2_, 60mM KCl, 0.4% NP-40, 10% glycerol, complete EDTA-free (Roche, 11873580001) and 10mM NEM). The cell extracts were homogenized by sonication for 5 s. NP-40 soluble and insoluble fractions were separated by centrifugation at 14,000 RPM for 20 min at 4 °C. Whole cell lysates (precentrifugation), soluble (supernatant, postcentrifugation), and insoluble fractions (pellet fraction and postcentrifugation) were captured, supplied with SDS, boiled and resolved on SDS–PAGE.

### Filter trap assay

To determine protein aggregation, the filter trap assay was performed^34^. Briefly, cell lysates were made one day after transfection in FTA buffer (10mM Tris-Cl, 150mM NaCl; pH = 8.0) containing 2% SDS. Serial dilutions of the protein extracts (typically 1:5 and 1:25) were applied onto a 0.2μm pore cellulose–acetate membrane prewashed with FTA-buffer containing 0.1% SDS. Aggregated proteins trapped in the membrane were immuno-detected using mouse anti-GFP antibody JL-8 (Clontech) and visualized as in WB.

### Western blotting

Following the preparation of protein samples, proteins were resolved by SDS-PAGE, transferred to nitrocellulose membrane and processed for WB. Primary antibodies (at concentrations mentioned above) were prepared in 3% BSA/PBS-Tween, secondary antibodies (Invitrogen, horse peroxidase conjugated IGG or IGM) in 5% milk/PBS-Tween. For visualization membranes were incubated with ECL western blotting substrate (Pierce, cat. No. 32106) for 2 min and developed (Sigma, cat. No. P7042-1GA) and fixed (Sigma, cat. No. P7167-1GA) using luminescent films (Amersham hyperfilm, GE-healthcare, cat. No. 28906837). Quantification of western blots was done using Image lab 5.0 software.

### Immunofluorescence

HeLa cells were seeded on poly-d-lysine coated glass slides and fixed 48 h posttransfection. HeLa cells were washed once with PBS (Gibco) and fixed with 2% formaldehyde (Sigma-Aldrich, St. Louis, MO, USA) for 15 min at room temperature. Cells were washed with PBS and PBS+ (PBS with 0.5% BSA and 0.15% glycine)^34,49^. Primary antibody incubation was performed overnight at 4 °C in a humid chamber and Alexa-conjugated dyes (Invitrogen, Carlsbad, CA, USA; Alexa Fluor 488 goat-anti-Rabbit, Alexa Fluor 488 donkey-anti-Rabbit, Alexa Fluor 594 goat-anti-Rabbit, Alexa Fluor 594 donkey-anti-Rabbit, Alexa Fluor 488 goat-anti-mouse, Alexa Fluor 594 goat-anti-mouse, and Alexa Fluor 594 chicken-anti-rat) were applied for 1.5 h at room temperature to visualize primary antibodies. Nuclear counterstaining was performed with 4′,6-diamidino-2-phenylindole (DAPI; Invitrogen) or Hoechst 33258 for 5 min, and samples were and embedded in glycerol (CitiFluor, Agar Scientific). LHCNM2 cells were grown on poly-l-lysine coated glass coverslip. 24 h after seeding, cells were transfected using lipofectamine 3000 according to manufacturer protocol. After 24 h transfection, cells were fixed with 3.7% formaldehyde for 9 min at room temperature, permeabilized with cold (−20 °C) acetone for 5 min at −20 °C. Cells were rinsed with PBS 1× and processed for immunostaining.

### Imaging

IF images were captured using confocal laser scanning microscope (Leica TCS SP8) with a 63×/1.40 objective lens. Z-stack images were obtained to check for the aggregates in different Z-planes. Quantification of the aggregates in various mutants was carried out manually using Leica DM6000 microscope. Imaris, photoshop, and Image J software was used for image processing.

### Clinical data

Standard protocol approvals, registrations, and patient consents. Clinical data and sample collection: The families were seen by one of the authors (S.S.S. or C.C.W.) in an outpatient clinic, where clinical neurophysiology was also performed.

### Clinical data and sample collection

IRB approval was obtained from the University of Pennsylvania for these studies. Written informed consent was obtained from each patient that participated. Each family member was seen by one of the authors (S.S.S., or C.C.W.) in an outpatient clinic, where clinical neurophysiology was performed with standard methods.

### Whole-exome sequencing and analysis

Genomic DNA was isolated from peripheral blood from all participants. Exome DNA was captured using the SureSelect, Human All Exon5 50 Mb kit (Agilent, Stanta Clara, CA) and sequenced on a HiSeq 2000 (Illumina, San Diego, CA). Paired-end reads of 100 bp length were generated and alignment and variant calls were made using BWA^51^ and GATK software packages^50^. Data were then imported into GEM.app, a web-based collaborative genome analysis tool^52^, where variants were filtered for de novo non-synonymous or splice site variants with frequency in public databases (MAF < 0.01 in NHLBI EVS), conservation (GERP > 2, PhastCons Score > 0.5, or phyloP Score > 0.75). The BAG3 variant was confirmed by bidirectional Sanger sequencing using forward (CCCAAGAGTGTGGCTACAGAA) and reverse (GTGTGGGGATCTTCTGCATT) primers.

## Supplementary information

Supplementary Information

Description of Additional Supplementary Files

Supplementary Movie 1

Reporting Summary

Source Data

## Data Availability

The datasets and other data generated during the current study are available from the corresponding authors upon reasonable request. A Reporting Summary for this Article is available as a Supplementary Information file. The source data underlying the figures and Supplementary figures are provided as Source Data file.
