# Supplementary material for: Myopathy associated BAG3 mutations lead to protein aggregation by stalling Hsp70 networks
Source: Nat Commun. 2018 Dec 17;9:5342. doi: 10.1038/s41467-018-07718-5 (PMC6297355; doi:10.1038/s41467-018-07718-5)
Supplement: Supplementary file 1 — Supplementary Information [file 41467_2018_7718_MOESM1_ESM.pdf]

Myopathy associated BAG3 mutations lead to protein aggregation by stalling Hsp70 networks

M.Meister-Broekema, R. Freilich, C. Jagadeesan, J.N. Rauch et al.

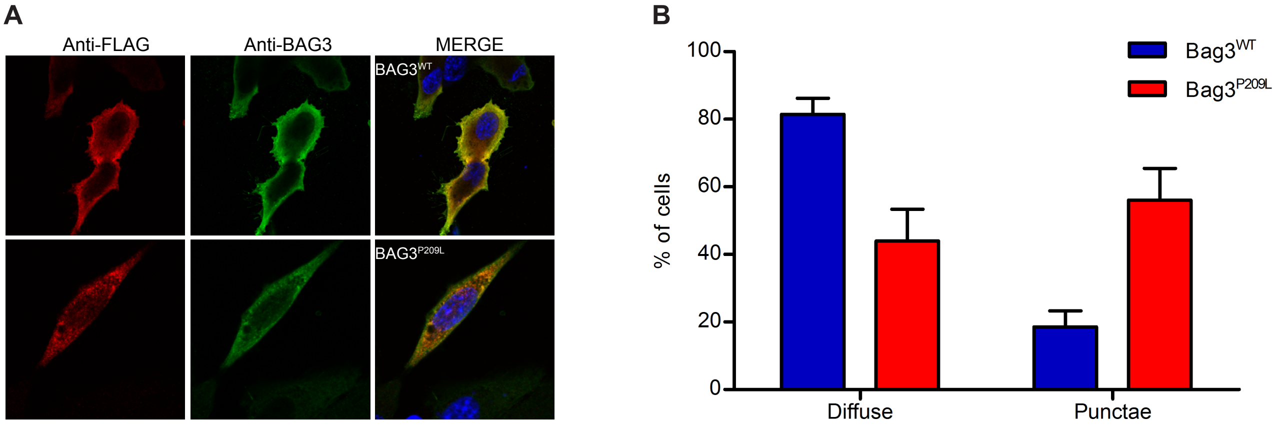

Supplementary Figure 1: **a** Immunofluorescence pictures of myoblast expressing FLAG-BAG3WT or FLAG-BAG3P209L, using BAG3 (green) or FLAG (red) antibodies. DAPI staining is shown in blue. **b** Quantification of punctae and diffuse staining of BAG3 in myoblast expressing BAG3WT or BAG3P209L. Error bars represent the SD. The number of cells with punctae is significantly increased in cells expressing BAG3P209L with a p-value of >0.05 (using Posthoc test: Bonferroni-Holm). Source data are provided as a Source data file.

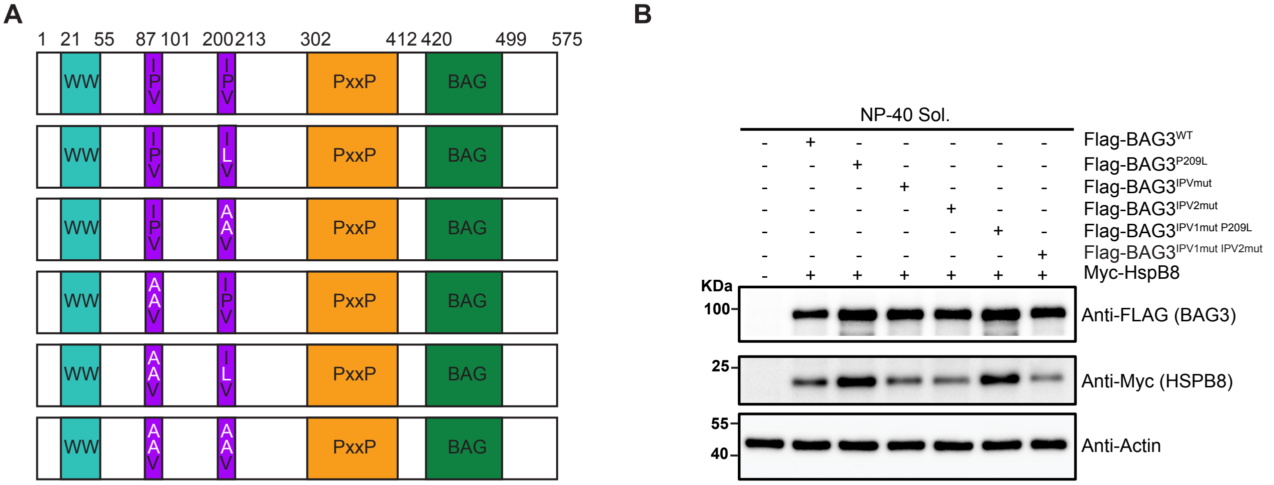

Supplementary Figure 2: **a** Schematic representation of BAG3 mutants used. **b** NP-40 soluble fractions belonging to Figure 2C of cells expressing indicated FLAG-BAG3 variants. Western blot against the indicated antibodies is shown. Source data are provided as a Source data file.

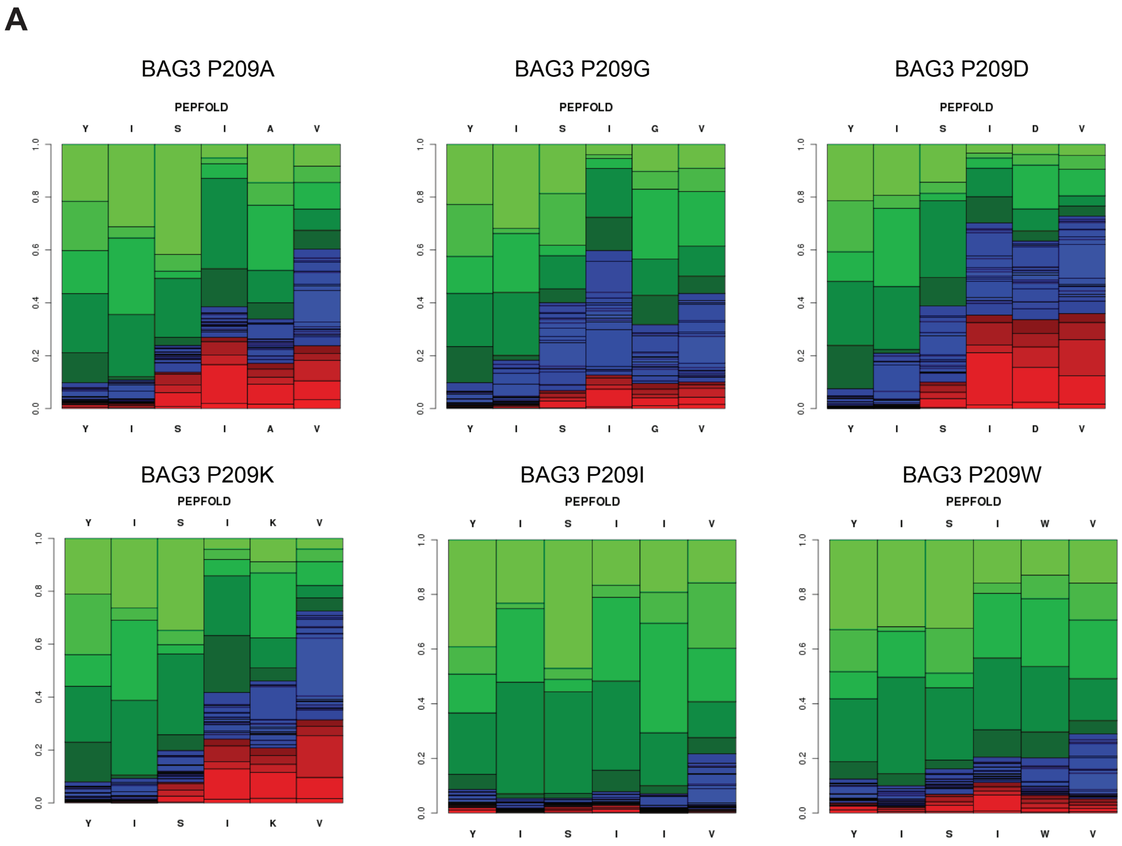

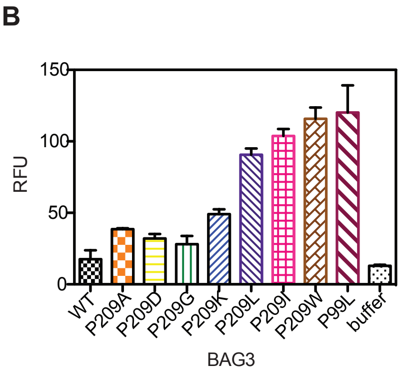

C

mutant	Hsp27c affinity (fold change)	N
controls		
P209A	2.0	1.9
P209D	1.9	1.9
P209G	1.9	1.8
P209K	2.6	2.0
hydrophobics		
P209I	3.6	0.9
P209L	3.7	1.1
P209W	2.8	1.3

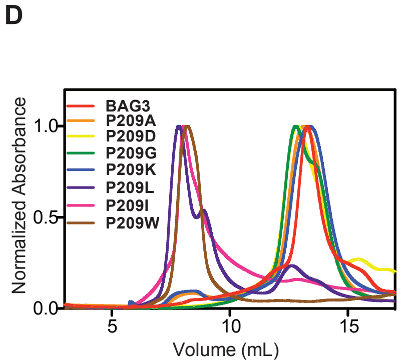

mutant	peak rt (min)	monomer %
controls		
WT	13.3	100%
P209A	12.8	93%
P209D	13.2	98%
P209G	12.7	89%
P209K	13.4	100%
hydrophobics		
P209I	8.1	3%
P209L	7.8	19%
P209W	8.1	11%

Supplementary Figure 3: **a** PepFold predictions for additional hydrophobic and control mutants at position 209. **b** Hydrophobic BAG3 mutants, but not controls, bind to ThT in vitro. Results are the average of at least three independent experiments performed in triplicate each. Error bars represent SD. Relative ThT fluorescence is shown. **c** Normalized (to BAG3WT) affinity and stoichiometric ratio (N) for Hsp27c/HSPB1c of indicated BAG3 mutants. **d** BAG3P209L and hydrophobic BAG3 mutants, but not controls, are soluble oligomer, as observed by SEC-MALS. Protein solutions (230 μM) were separated by SEC and the size of particles estimated by light scattering. Retention times and percent monomer are shown. Results are representative of measurements performed in triplicate. Source data are provided as a Source data file.

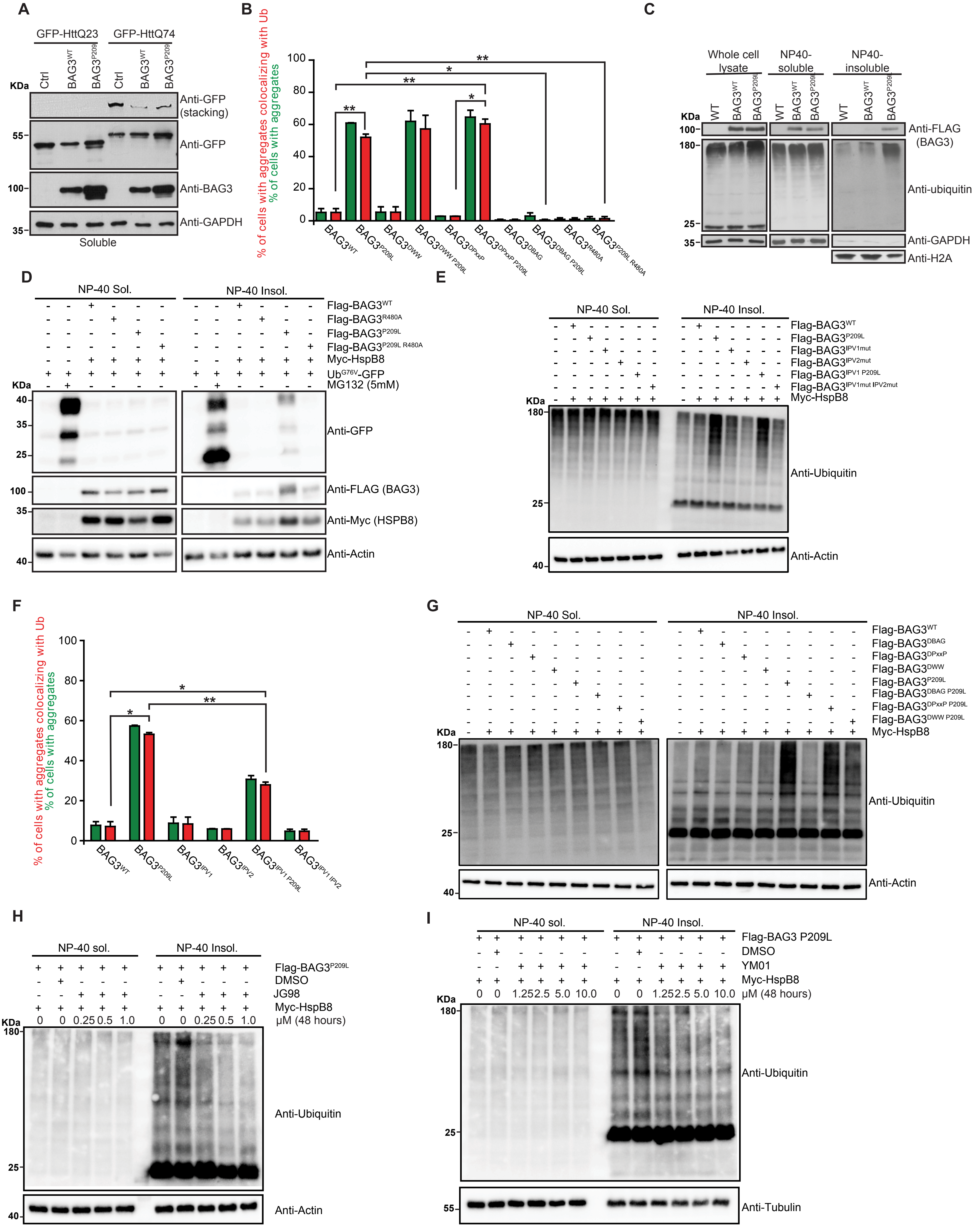

A
GFP-HttQ23
GFP-HttQ74
Ctrl
BAG3WT
BAG3P209L
KDa
Anti-GFP (stacking)
55
Anti-GFP
100
Anti-BAG3
35
Anti-GAPDH
Soluble
B
% of cells with aggregates colocalizing with Ub
% of cells with aggregates
100
80
60
40
20
0
**
*
BAG3WT
BAG3P209L
BAG3DWW
BAG3DWW P209L
BAG3DPxxP
BAG3DPxxP P209L
BAG3DBAG
BAG3DBAG P209L
BAG3R480A
BAG3P209L R480A
C
Whole cell lysate
NP40-soluble
NP40-insoluble
WT
BAG3WT
BAG3P209L
KDa
100
180
25
35
Anti-FLAG (BAG3)
Anti-ubiquitin
Anti-GAPDH
Anti-H2A
D
NP-40 Sol.
NP-40 Insol.
Flag-BAG3WT
Flag-BAG3R480A
Flag-BAG3P209L
Flag-BAG3P209L R480A
Myc-HspB8
UbG76V-GFP
MG132 (5mM)
KDa
40
35
25
100
Anti-GFP
Anti-FLAG (BAG3)
Anti-Myc (HSPB8)
Anti-Actin
E
NP-40 Sol.
NP-40 Insol.
Flag-BAG3WT
Flag-BAG3P209L
Flag-BAG3IPV1mut
Flag-BAG3IPV2mut
Flag-BAG3IPV1 P209L
Flag-BAG3IPV1mut IPV2mut
Myc-HspB8
KDa
180
25
40
Anti-Ubiquitin
Anti-Actin
F
% of cells with aggregates colocalizing with Ub
% of cells with aggregates
100
80
60
40
20
0
*
**
BAG3WT
BAG3P209L
BAG3IPV1
BAG3IPV2
BAG3IPV1 P209L
BAG3IPV1 IPV2
G
NP-40 Sol.
NP-40 Insol.
Flag-BAG3WT
Flag-BAG3DBAG
Flag-BAG3DPxxP
Flag-BAG3DWW
Flag-BAG3P209L
Flag-BAG3DBAG P209L
Flag-BAG3DPxxP P209L
Flag-BAG3DWW P209L
Myc-HspB8
KDa
180
25
40
Anti-Ubiquitin
Anti-Actin
H
NP-40 sol.
NP-40 Insol.
Flag-BAG3P209L
DMSO
JG98
Myc-HspB8
0 0 0.25 0.5 1.0
μM (48 hours)
KDa
180
25
40
Anti-Ubiquitin
Anti-Actin
I
NP-40 sol.
NP-40 Insol.
Flag-BAG3 P209L
DMSO
YM01
Myc-HspB8
0 0 1.25 2.5 5.0 10.0
μM (48 hours)
KDa
180
25
55
Anti-Ubiquitin
Anti-Tubulin

Supplementary Figure 4: **a** Effect of FLAG-BAG3WT or FLAG-BAG3P209L on expression levels of GFP-HttQ23 and GFP-HttQ74 (exon-1 of the huntingtin gene) and on GFP-HttQ74 aggregation (stacking). Western blot against indicated antibodies is shown (belongs to Figure 6D,E). **b** Quantification of the percentage of HeLa cells with BAG3 aggregates (green) and percentage of cells with BAG3 aggregates that co-localize with ubiquitin (red). Cells expressed HSPB8 and BAG3 or the indicated variants of BAG3. Data represents the mean and standard deviation of two independent experiments at least 100 cells were counted per experiment (Welch t-test was used to calculate the P values,* indicates $P < 0.05$ and ** indicates $P < 0.01$). **c** Fractionation of HEK293 cells expressing a control, FLAG-BAG3WT or FLAG-BAG3P209L. Western blot against FLAG (BAG3), ubiquitin, GAPDH and H2A are shown. **d** Fractionation of stably expressing UbG76V-GFP HEK293 cells expressing HSPB8, a control or BAG3WT or BAG3P209L. Western blot against GFP, FLAG (BAG3), Myc (HSPB8) and tubulin is shown. **e** Fractionation of HEK293 cells expressing a control, FLAG-BAG3WT or various FLAG-BAG3 –IPV mutants as indicated. Western blot against FLAG (BAG3), ubiquitin and actin are shown. **f** Quantification of the percentage of HeLa cells with BAG3 aggregates (green) and percentage of cells with BAG3 aggregates that co-localize with ubiquitin (red). Cells expressed HSPB8 and BAG3 or the indicated variants of BAG3. Data represents the mean and standard deviation of two independent experiments at least 100 cells were counted per experiment (Welch t-test was used to calculate the P values,* indicates $P < 0.05$ and ** indicates $P < 0.01$). **g** Fractionation of HEK293 cells expressing HSPB8, a control or BAG3WT or BAG3P209L or the indicated single or double deletion mutants. Western blot against indicated antibodies is shown. The same samples as in Figure 5C have been used, loading control is therefore the same. **h** Fractionation of HEK293 cells expressing HSPB8 and BAG3P209L in the presence of increasing concentration of JG98. Western blot against indicated antibodies is shown. The same samples as in Figure 5G have been used, loading control is therefore the same. **i** Fractionation of HEK293 cells expressing HSPB8 and BAG3P209L in the presence of increasing concentration of YM01. Western blot against indicated antibodies is shown. The same samples as in figure 5H have been used, loading control is therefore the same. Source data are provided as a Source data file.

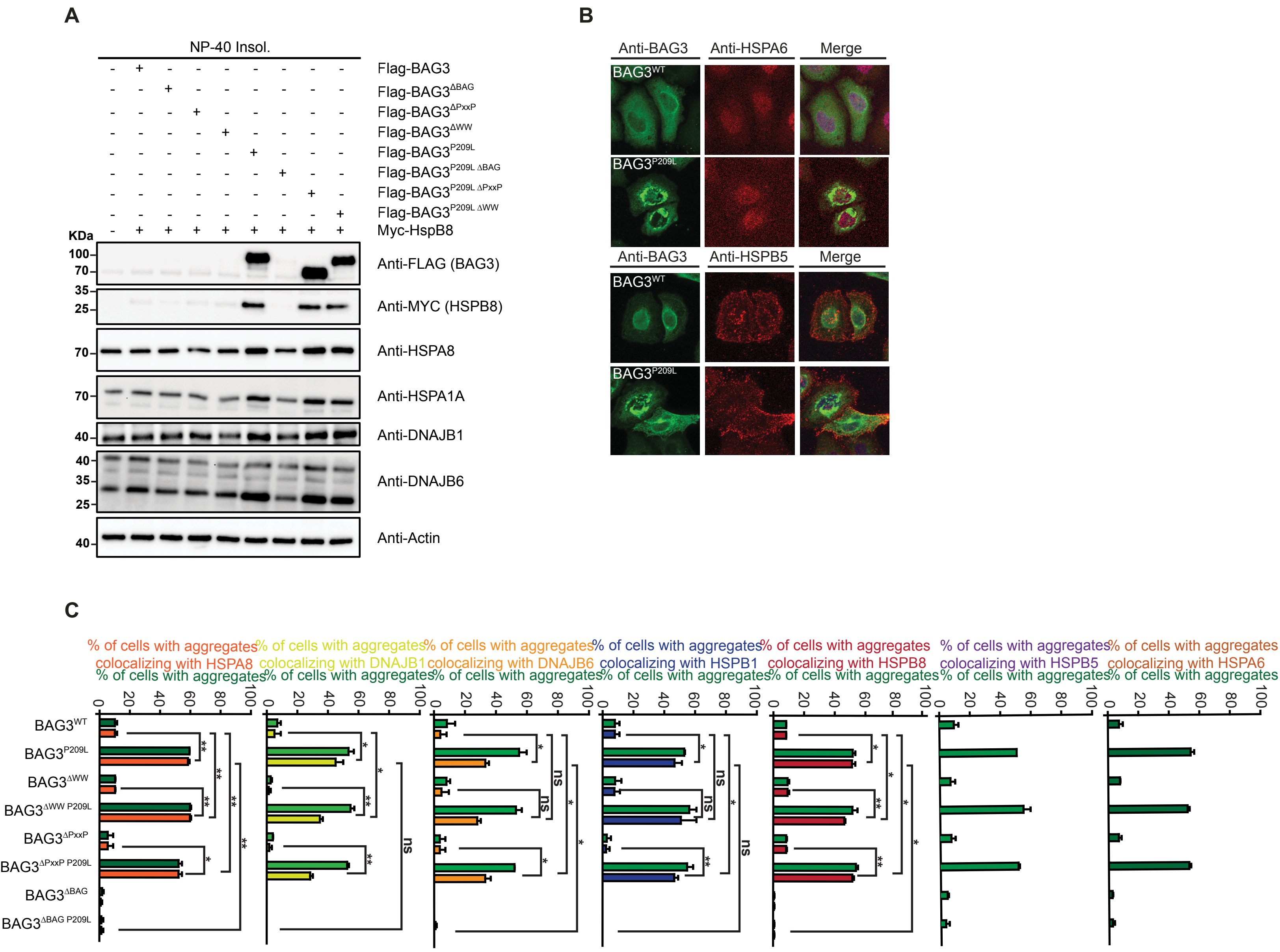

Supplementary Figure 5: **a** Fractionation of HEK293 cells expressing Myc-HSPB8 and BAG3 variants. Western blots using the indicated HSP antibodies are shown (additional data to Figure 7A). The same samples as in Figure 5C have been used, loading controls are therefore the same. **b** Immunofluorescence pictures of HeLa cells expressing FLAG-BAG3WT or FLAG-BAG3P209L. Cells were stained with the indicated antibodies for staining endogenous chaperones. **c** Quantification of the percentage of cells with aggregates and cells with aggregates that colocalize with the indicated chaperones. Data represents the mean and standard deviation of two independent experiments (at least 100 cells were counted per experiment, Welch t-test was used to calculate the P values, * indicates $P < 0.05$ and ** indicates $P < 0.01$ and ns is not significant). Source data are provided as a Source data file.

A

	Patient 1	Patient 2
**BAG3**	c.1408C>T<br>p.Pro407Ser	c.1408C>T<br>p.Pro407Ser
**Sex**	Male	Female
**Age**	45	37
**Onset**	35	28
**Weakness**	Symmetric	Mild asymmetry
**Deltoid**	4+	5
**Biceps**	5	5
**Triceps**	5	5
**Wrist extensors**	5	5
**Hand intrinsics**	5	5
**Iliopsoas**	4	5
**Quadriceps**	5	5
**Hamstrings**	5	5
**Gastrocnemius**	5	4-
**Tibialis anterior**	4	2-3
**Sensation**	NL	NL
**Reflexes**	Trace throughout	Absent at the ankles
**Creatine kinase**	618 (nl 30-200)	306 (nl 30-200)
**EMG**	Myopathic changes in proximal muscles and neurogenic features in distal leg muscles.	Acute and chronic denervation in the peroneal and tibial nerve innervated muscles and moderate to chronic denervation in the vastus.
**Biopsy**	Quadriceps and Deltoid with small angular fibers, rimmed vacuoles and TDP-43 inclusions<br>**RV myopathy.**	Fiber atrophy with centrally located eosinophilic and trichome positive inclusions, RVs and Z-disc streaming on EM. **Myobibrillar myopathy**

B

```
                                     470       480       490       500
BAG3 [Homo sapiens]          -----------LDSVD----PEGRADVRQARRDGVRKVQ--TILEKLEQKAIDVPGQVQV
BAG1 [Homo sapiens]          VKATIEQFMKILEEIDTLILPENFKDSRLKRKGLVKKVQ--AFLAECDTVEQNICQETE-
BAG2 [Homo sapiens]          -----------VETIR--N-PQQQESLKHATRIIDEVVN--KFLDDLGNAKSHLMSLYSA
BAG4 [Homo sapiens]          -----------LDSVE----TGGQDSVRQARKEAVCKIQ--AILEKLEKKGL--------
BAG5 [Homo sapiens]          -----------LDAVD----PQGEEKCKAARKQAVRLAQ--NILSYLDLKSDEWEY----
BAG6 [Homo sapiens]          ASASAGTTNT-ATTAG-PA-PGGPAQPPPTPQPSMADLQFSQLLGNLLGPAGPGAGGSGV
BAG3 [Mouse musculus]        -----------LDSVD----PEGRADVRQARRDGVRKVQ--TILEKLEQKAIDVPGQVQV
BAG3 [Danio rerio]           -----------LDSVD----PEGRPDVRQARRDGVRRVQ--NILDELEMIGEMQAGNEAK
BAG3 [Rattus norvegicus]     -----------LDSVD----PEGRADVRQARRDGVRKVQ--TILEKLEQKAIDVPGQVQV
BAG3 [Bos taurus]            -----------LDSVD----PEGRADVRQARRDGVRKVQ--TILEKLEQKAIDVPGQVQV
BAG3 [Macaca mulatta]        -----------LDSVD----PEGRADVRQARRDGVRKVQ--TILEKLEQKAIDVPGQVQV
```

Supplementary Figure 6: a Patient characteristics. **b** Alignment of BAG3 proteins show the high conservation of proline at position 470.

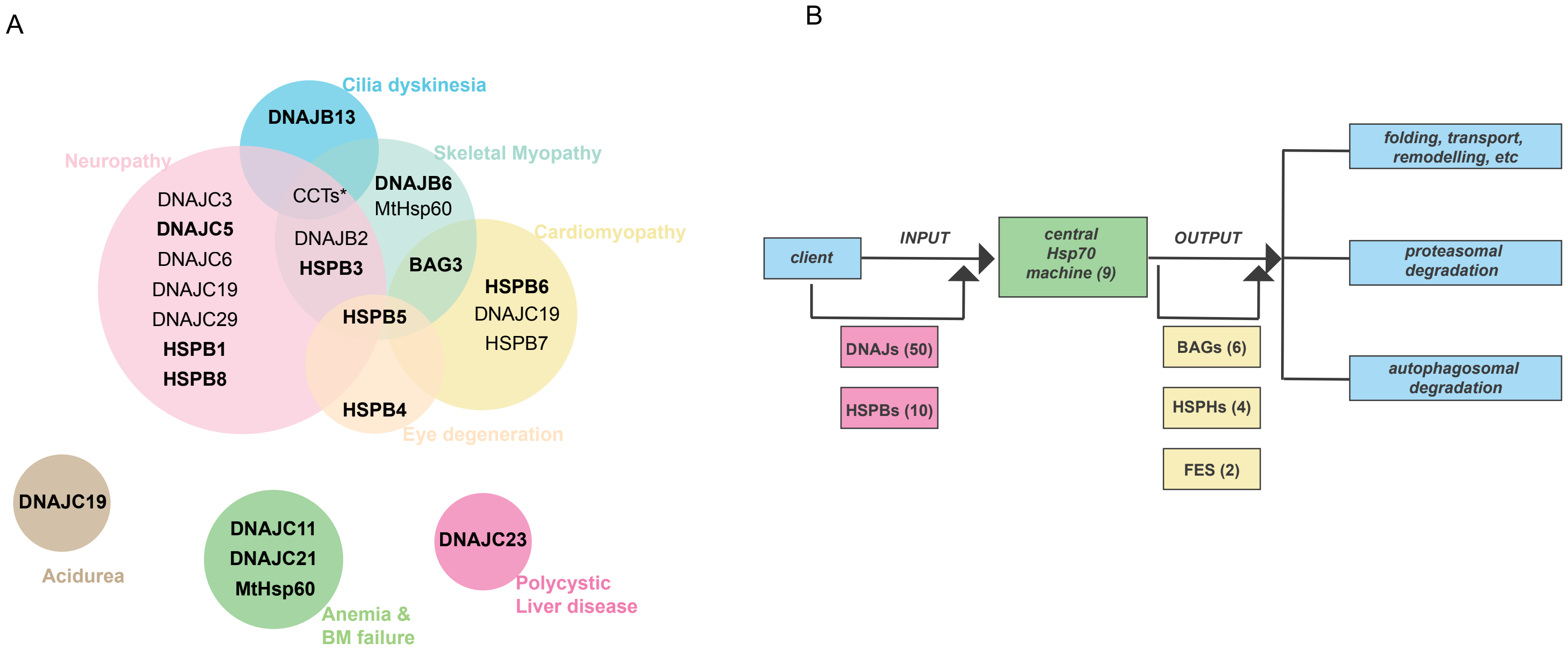

Supplementary Figure 7: Chaperonopathies. **a** Overview of chaperones for which mutations have been demonstrated to cause human dominant (bold) or recessive (non-bold) diseases. Note the over-representation of diseases in tissues with low turnover rates). It is important to also note that many but not all chaperonopathies, especially the recessive forms, are associated with protein aggregation. **b** Basic Hsp70 machines existing of the central Hsp70 proteins (9 isoforms in humans) and regulation of client input by the 50 different human DNAJs and 10 HSPBs; Client output (fate) is in part regulated by the different nucleotide exchange factors (NEFs), including HSPHs (4 in humans), BAG6 (6 in humans) and FES-like proteins (2 in humans). Note, the fate of the client is likely also largely determined by its folding state and that certain DNAJs/HSPBs may already co-determine fate, e.g. via interactions with specific NEFS (not indicated).

Supp. Table I.

Primers used for cloning *E.coli* strains in this study

Primer	Sequence
Bag3 EcoRI F	TACTACGAATTCTATGAGCGCCGCCACCCACTCGC
bag3_reverse_XhoI	GTAGTACTCGAGCTACGGTGCTGCTGGGTTACCAGG
BAG3P209L For	GGTACATCTCCATTCTGGTGATACACGAGC
BAG3P209L Rev	GCTCGTGTATCACCAGAATGGAGATGTACC
Bag3 dWW For	CCCAAGGAGACTCCATCCTCTGCCAATG
Bag3 dWW Rev	CAAAGGGTCGCGGTCACCGTTGCCG
dBag VtoA For	CATGATGCAGGTGGCGTCCGGCAACGGTG
dBag VtoA Rev	CACCGTTGCCGGACGCCACCTGCATCATG
Bag3 dIPV1 for	CCAGGCTACATTGCCGCTGCTGTGCTCCATGAAG
Bag3 dIPV1 rev	CTTCATGGAGCACAGCAGCGGCAATGTAGCCTGG
Bag3 dIPV2 for	GTACATCTCCGCTGCGGTGATACACGAGC
Bag3 dIPV2 rev	GCTCGTGTATCACCGCAGCGGAGATGTAC
CMV For	CGCAAATGGGCGGTAGGCGT
Rev CMV	ACAGTGGGAGTGGCACCTTC
Bag3 nt623	CGGTGATACACGAGCAGAAC
T7 promoter For	TAATACGACTCACTATAGG

Primers used for cloning BAG3 and its mutants.

BAG3 cloning primers (into pMCSG7 using LIC cloning) 5’-> 3’:

Fwd: TACTTCCAATCCAATGCAatgagcgccgccacccactcgcccatgat
Rev: TTATCCACTTCCAATGTTActacggtgctgctgggttaccaggggtgtctgtc

Mutagenesis primers:

P209L

Fwd: gggtacatctccattctggtgatacacgagcagaa

Rev: ccgggtaacgttctgctcgtgtatcaccagaatggag

P209I

Fwd: gggtacatctccattatcgtgatacacgagcagaa

Rev: ccgggtaacgttctgctcgtgtatcacgataatggag

P209A

Fwd: gggtacatctccattgcggtgatacacgagcagaa

Rev: ccgggtaacgttctgctcgtgtatcaccgcaatggag

P209G

Fwd: gggtacatctccattggggtgatacacgagcagaa

Rev: ccgggtaacgttctgctcgtgtatcaccccaatggag

P209W

Fwd: gggtacatctccatttgggtgatacacgagcagaa

Rev: ccgggtaacgttctgctcgtgtatcacccaaatggag

P209D

Fwd: gggtacatctccattgacgtgatacacgagcagaa

Rev: ccgggtaacgttctgctcgtgtatcacgtcaatggag

P209K

Fwd: gggtacatctccattaaggtgatacacgagcagaa

Rev: ccgggtaacgttctgctcgtgtatcaccttaatggag

P99L

Fwd: caggctacattcccattcttgtgctccatgaag

Rev: gcgccttcatggagcacaagaatgggaatgtag

IPV1mut:

Fwd: gctacattcccggtcctgggctccatgaaggcgct

Rev: cttcatggagcccaggaccgggaatgtagcctggt

IPV2mut:

Fwd: gtacatctccggtccggggatacacgagcagaa

Rev: gtatccccggaccggagatgtacccccgcg

Supplementary Table S2. Plasmids used in this study

Plasmid	Description	Reference
pMCSG7 BAG3	pMCSG7 containing His tagged, TEV protease cleavable BAG3, Amp^R	Rauch et al. 2014[24]
pMCSG7 BAG3 mutants	pMCSG7 containing His tagged, TEV protease cleavable BAG3 P209L/A/G/D/K/I/W or IPV1/IPV2/IPV12, Amp^R	This study
pMCSG7 Hsp27c	pMCSG7 containing His tagged, TEV protease cleavable BAG3, Amp^R	Rauch et al. 2014[21]
pMCSG7 Hsc70/HSPA8	pMCSG7 containing full length Hsc70 or $Hsc70^{NBD}$, His tagged and TEV protease cleavable, Amp^R	Rauch et al. 2014[21]
pcDNA3 FLAG-Parkin	pcDNA containing FLAG tagged human Parkin, Amp^R	Christian Behl
pCIN His-BAG3	pCINeo containing His tagged human BAG3, Amp^R	Serena Carra
pCIN His-$BAG3^{\Delta BAG}$	pCINeo containing His tagged human BAG3 with BAG domain deletion, Amp^R	Serena Carra
pCIN His-$BAG3^{\Delta PxxP}$	pCINeo containing His tagged human BAG3 with PxxP domain deletion, Amp^R	Serena Carra
pCIN Myc-HspB8	pCINeo containing Myc tagged human HspB8, Amp^R	Serena Carra
pcDNA3 FLAG-BAG3	pcDNA3 containing FLAG tagged human BAG3, Amp^R	This study
pcDNA3 FLAG-$BAG3^{P209L}$	pcDNA3 containing FLAG tagged human BAG3 with P209L point mutation, Amp^R	This study
pcDNA3 FLAG-$BAG3^{\Delta BAG}$	pcDNA3 containin FLAG tagged human BAG3 with BAG domain deletion, Amp^R	This study
pcDNA3 FLAG-$BAG3^{\Delta BAG\ P209L}$	pcDNA3 containing FLAG tagged human BAG3 with BAG domain deletion and P209L point mutation, Amp^R	This study
pcDNA3 FLAG-$BAG3^{\Delta PxxP}$	pcDNA3 containing FLAG tagged human BAG3 with PxxP domain deletion, Amp^R	This study
pcDNA3 FLAG-$BAG3^{\Delta PxxP\ P209L}$	pcDNA3 containing FLAG tagged human BAG3 with PxxP domain deletion and P209L point mutation, Amp^R	This study
pcDNA3 FLAG-$BAG3^{\Delta WW}$	pcDNA3 containing FLAG tagged human BAG3 with WW domain deletion, Amp^R	This study
pcDNA3 FLAG-$BAG3^{\Delta WW\ P209L}$	pcDNA3 containing FLAG tagged human BAG3 with WW domain deletion and P209L point mutation, Amp^R	This study
pcDNA3 FLAG-$BAG3^{R480A}$	pcDNA3 containing FLAG tagged human BAG3 with R480A point mutation, Amp^R	This study
pcDNA3 FLAG-$BAG3^{P209L\ R480A}$	pcDNA3 containing FLAG tagged human BAG3 with P209L and R480A point mutations, Amp^R	This study
pcDNA3 FLAG-$BAG3^{IPV1mut}$	pcDNA3 containing FLAG tagged human BAG3 with IPV1 substituted to AAA, Amp^R	This study
pcDNA3 FLAG-$BAG3^{IPV2mut}$	pcDNA3 containing FLAG tagged human BAG3 with IPV2 substituted to AA, Amp^R	This study

pcDNA3 FLAG-BAG3$^{IPV1mut-P209L}$	pcDNA3 containing FLAG tagged human BAG3 with IPV1 substituted to AAA and P209L point mutation, Amp^{R}	This study
pcDNA3 FLAG-BAG3$^{IPV1mut-IPV2\ mut}$	pcDNA3 containing FLAG tagged human BAG3 with IPV1 substituted to AAA and IPV2 substituted to AA, Amp^{R}	This study

Supplementary methods

Additional information recombinant protein production

Briefly, HSPBs and Hsp70s were expressed in BL21(DE3) cells and single colonies were used to inoculate TB medium containing ampicillin (50 µg/mL). Cultures were grown at 37 °C for 5 hours, cooled to 20 °C and induced overnight with 200 µM IPTG. Hsp72 variants were purified using a His column, followed by cleavage of the tag via TEV protease, and final purification on an ATP-agarose column. Briefly, proteins were first purified by batch purification with Ni-NTA His•Bind® Resin (Novagen, Darmstadt, Germany) following the user manual. The His-tag of eluted protein was then removed by His-tagged TEV protease (1 mM DTT, 4 °C, overnight incubation). After adjusting the $MgCl_2$ and KCl concentration to 10 mM, the sample was further purified by ATP-agarose column. Finally, the remaining cleaved His-tag was removed by Ni-NTA column. Hsp27c is tagless, so it was purified using a two-step ammonium sulfate precipitation followed by MonoQ and SEC. Briefly, ammonium sulfate was added to a final concentration of 16.9% (w/v), centrifuged, pellet discarded, and then an additional 16.9% (w/v) ammonium sulfate was added to the supernatant to precipitate the protein from solution. Precipitated protein was brought up and dialyzed into MonoQ Buffer A (20 mM Tris, pH 8.0) overnight, followed by a MonoQ column (0-1 M NaCl gradient), and finally an SEC on a Superdex S75 (Hsp27c) or Superdex S200 (GE Healthcare) equilibrated in 50 mM sodium phosphate, 100 mM NaCl, pH 7.5 buffer.
