# Supplementary material for: Myopathy associated BAG3 mutations lead to protein aggregation by stalling Hsp70 networks
Source: Nat Commun. 2018 Dec 17;9:5342. doi: 10.1038/s41467-018-07718-5 (PMC6297355; doi:10.1038/s41467-018-07718-5)
Supplement: Supplementary file 2 — Description of Additional Supplementary Files [file 41467_2018_7718_MOESM2_ESM.pdf]

Description of Additional Supplementary Files

File Name: Supplementary Movie 1
Description: 3D reconstruction of HeLA cells expressing FLAG-BAG3P209L, cells we restained with BAG3 (green) and LaminA/C (red) antibodies and with DAPI (blue).
