# Supplementary material for: Myopathy associated BAG3 mutations lead to protein aggregation by stalling Hsp70 networks
Source: Nat Commun. 2018 Dec 17;9:5342. doi: 10.1038/s41467-018-07718-5 (PMC6297355; doi:10.1038/s41467-018-07718-5)
Supplement: Supplementary file 4 — Reporting Summary [file 41467_2018_7718_MOESM4_ESM.pdf]

nature research

Corresponding author(s): Steven Bergink, Jason E. Gestwicki, Harm H. Kampinga

Reporting Summary

Nature Research wishes to improve the reproducibility of the work that we publish. This form provides structure for consistency and transparency in reporting. For further information on Nature Research policies, see Authors & Referees and the Editorial Policy Checklist.

Statistical parameters

When statistical analyses are reported, confirm that the following items are present in the relevant location (e.g. figure legend, table legend, main text, or Methods section).

n/a	Confirmed	
☐	☒	The exact sample size (*n*) for each experimental group/condition, given as a discrete number and unit of measurement
☐	☒	An indication of whether measurements were taken from distinct samples or whether the same sample was measured repeatedly
☐	☒	The statistical test(s) used AND whether they are one- or two-sided<br>*Only common tests should be described solely by name; describe more complex techniques in the Methods section.*
☒	☐	A description of all covariates tested
☒	☐	A description of any assumptions or corrections, such as tests of normality and adjustment for multiple comparisons
☐	☒	A full description of the statistics including central tendency (e.g. means) or other basic estimates (e.g. regression coefficient) AND variation (e.g. standard deviation) or associated estimates of uncertainty (e.g. confidence intervals)
☒	☐	For null hypothesis testing, the test statistic (e.g. *F*, *t*, *r*) with confidence intervals, effect sizes, degrees of freedom and *P* value noted<br>*Give P values as exact values whenever suitable.*
☒	☐	For Bayesian analysis, information on the choice of priors and Markov chain Monte Carlo settings
☒	☐	For hierarchical and complex designs, identification of the appropriate level for tests and full reporting of outcomes
☒	☐	Estimates of effect sizes (e.g. Cohen's *d*, Pearson's *r*), indicating how they were calculated
☐	☒	Clearly defined error bars<br>*State explicitly what error bars represent (e.g. SD, SE, CI)*

Our web collection on statistics for biologists may be useful.

Software and code

Policy information about availability of computer code

Data collection	n/a
Data analysis	n/a

For manuscripts utilizing custom algorithms or software that are central to the research but not yet described in published literature, software must be made available to editors/reviewers upon request. We strongly encourage code deposition in a community repository (e.g. GitHub). See the Nature Research guidelines for submitting code & software for further information.

Data

Policy information about availability of data

All manuscripts must include a data availability statement. This statement should provide the following information, where applicable:

- Accession codes, unique identifiers, or web links for publicly available datasets
- A list of figures that have associated raw data
- A description of any restrictions on data availability

Provide your data availability statement here.

Field-specific reporting

Please select the best fit for your research. If you are not sure, read the appropriate sections before making your selection.

☒ Life sciences ☐ Behavioural & social sciences ☐ Ecological, evolutionary & environmental sciences

For a reference copy of the document with all sections, see nature.com/authors/policies/ReportingSummary-flat.pdf

Life sciences study design

All studies must disclose on these points even when the disclosure is negative.

Sample size	NA
Data exclusions	NA
Replication	experiments were repeated (n is indicated in the legends), all experiments were reproducible
Randomization	n/a
Blinding	n/a

Reporting for specific materials, systems and methods

Materials & experimental systems

n/a	Involved in the study	
☐	☒	Unique biological materials
☐	☒	Antibodies
☐	☒	Eukaryotic cell lines
☒	☐	Palaeontology
☒	☐	Animals and other organisms
☐	☒	Human research participants

Methods

n/a	Involved in the study	
☒	☐	ChIP-seq
☒	☐	Flow cytometry
☒	☐	MRI-based neuroimaging

Unique biological materials

Policy information about availability of materials

Obtaining unique materials	Unique material contains specific antibodies and patient-derived fibroblast. Request can be send to the corresponding authors. These items are in principle available, depending on the presence (since these are limited resources) and the type of request (e.g. academic or commercial)

Antibodies

Antibodies used	Antibodies (dilutions are indicated in brackets for western blot (WB), immunofluorescence (IF) or immunoprecipitation (IP)) against FLAG (Sigma, clone M2; Sigma, produced in Rabbit, IP 3ul/sample, IF 1:100, WB 1:1000), FLAG (Sigma, clone M2; Sigma, M, Wb 1:1000, IF 1:200), ubiquityl-histone H2A (Millipore, clone E6C5), ubiquitin (Norvus Biologicals, FK2, M, WB 1:1000, IF 1:1000; Dako WB), K48-linkage specific polyubiquitin (Enzo lifesciences, WB 1:1000), K63-linkage specific polyubiquitin (Cell Signalling, clone D7A11, 1:1000), myc (MBL, clone PL14, WB 1:3000, IF 1:100), HSC70 (Stressgen, WB 1:5000, IF 1:100), LC3B (Novus Biologicals, NB100-220), GFP (clonetech, 632381), p62 (Enzo Life Sciences, BML-PW9860), Lamin A/C (Santa Cruz, 4A58), HSC70 (Stressmarq biosciences), HSP70 (Stressgen, clone SPA-810, WB 1:1000, IF 1:50), HSPA1A (Enzo life sciences, Rb, WB 1:1000), HSPB1 (Stressmarq biosciences), GAPDH (Fitzgerald, clone 6C5, WB 1:50000), histone H2A (Abcam, WB 1:5000), MYC (Clonetech, Mountain View, CA, USA), DNAJB1/Hsp40 (Stressgen, San Diego, CA, USA, Rb, 1:1000) were used.
Validation	antibodies were validated by adding negative controls (i.e. sample that doesnt contain the epitope) or positive controls (i.e. samples that induce or reduce a certain epitope, as for example for the ubiquitin and ubiquitylated H2A antibodies). Not all of these controls are added to the manuscript. These controls are performed regularly.

Eukaryotic cell lines

Policy information about cell lines

Cell line source(s)	HeLa (human cervical cancer), HEK293, and HEK293T (human embryonal kidney) are standard cell lines<br>primary fibroblast are either described for the first time (IRB approval was obtained from the University of Pennsylvania for these studies. Written informed consent was obtained from each patient that participated. Each family member was seen by one of the authors (SSS, or CCW) in an outpatient clinic, where clinical neurophysiology was performed with standard methods.) or obtained from Telethon Genetic BioBank (GTB12001D to Elena Pegoraro) and Eurobiobank (the P209L patient-derived cell lines)
Authentication	primary fibroblast were authenticated by western blot and sequencing<br>HEK293 has been sequenced (and is confirmed), HeLa hasn't been authenticated
Mycoplasma contamination	all cell lines are regularly checked for (the absence of) mycoplasma contamination. All experiments were performed on Mycoplasma negative cell lines
Commonly misidentified lines<br>(See ICLAC register)	n/a

Human research participants

Policy information about studies involving human research participants

Population characteristics	n/a
Recruitment	IRB approval was obtained from the University of Pennsylvania for these studies. Written informed consent was obtained from each patient that participated. Each family member was seen by one of the authors (SSS, or CCW) in an outpatient clinic, where clinical neurophysiology was performed with standard methods.
